# Impact of disinvestment from weekend allied health services across acute medical and surgical wards: 2 stepped-wedge cluster randomised controlled trials

**DOI:** 10.1371/journal.pmed.1002412

**Published:** 2017-10-31

**Authors:** Terry P. Haines, Kelly-Ann Bowles, Deb Mitchell, Lisa O’Brien, Donna Markham, Samantha Plumb, Kerry May, Kathleen Philip, Romi Haas, Mitchell N. Sarkies, Marcelle Ghaly, Melina Shackell, Timothy Chiu, Steven McPhail, Fiona McDermott, Elizabeth H. Skinner

**Affiliations:** 1 Department of Physiotherapy, Monash University, Frankston, Victoria, Australia; 2 Allied Health Research Unit, Monash Health, Cheltenham, Victoria, Australia; 3 Department of Community Emergency Health and Paramedic Practice, Monash University, Frankston, Victoria, Australia; 4 Department of Occupational Therapy, Monash University, Frankston, Victoria, Australia; 5 Monash Medical Centre, Allied Health, Monash Health, Clayton, Victoria, Australia; 6 Royal Melbourne Hospital, Melbourne, Victoria, Australia; 7 Department of Health and Human Services, Melbourne, Victoria, Australia; 8 Department of Physiotherapy, Footscray Hospital, Western Health, Footscray, Victoria, Australia; 9 Footscray Hospital, Western Health, Footscray, Victoria, Australia; 10 Institute of Biomedical Innovation, Queensland University of Technology and Centre for Functioning and Health Research, Buranda, Queensland, Australia; 11 Department of Social Work, Monash Medical Centre, Monash Health and Monash University, Clayton, Victoria, Australia; Edinburgh University, UNITED KINGDOM

## Abstract

**Background:**

Disinvestment (removal, reduction, or reallocation) of routinely provided health services can be difficult when there is little published evidence examining whether the services are effective or not. Evidence is required to understand if removing these services produces outcomes that are inferior to keeping such services in place. However, organisational imperatives, such as budget cuts, may force healthcare providers to disinvest from these services before the required evidence becomes available. There are presently no experimental studies examining the effectiveness of allied health services (e.g., physical therapy, occupational therapy, and social work) provided on weekends across acute medical and surgical hospital wards, despite these services being routinely provided internationally. The aim of this study was to understand the impact of removing weekend allied health services from acute medical and surgical wards using a disinvestment-specific non-inferiority research design.

**Methods and findings:**

We conducted 2 stepped-wedge cluster randomised controlled trials between 1 February 2014 and 30 April 2015 among patients on 12 acute medical or surgical hospital wards spread across 2 hospitals. The hospitals involved were 2 metropolitan teaching hospitals in Melbourne, Australia. Data from *n =* 14,834 patients were collected for inclusion in Trial 1, and *n =* 12,674 in Trial 2. Trial 1 was a disinvestment-specific non-inferiority stepped-wedge trial where the ‘current’ weekend allied health service was incrementally removed from participating wards each calendar month, in a random order, while Trial 2 used a conventional non-inferiority stepped-wedge design, where a ‘newly developed’ service was incrementally reinstated on the same wards as in Trial 1. Primary outcome measures were patient length of stay (proportion staying longer than expected and mean length of stay), the proportion of patients experiencing any adverse event, and the proportion with an unplanned readmission within 28 days of discharge. The ‘no weekend allied health service’ condition was considered to be not inferior if the 95% CIs of the differences between this condition and the condition with weekend allied health service delivery were below a 2% increase in the proportion of patients who stayed in hospital longer than expected, a 2% increase in the proportion who had an unplanned readmission within 28 days, a 2% increase in the proportion who had any adverse event, and a 1-day increase in the mean length of stay. The current weekend allied health service included physical therapy, occupational therapy, speech therapy, dietetics, social work, and allied health assistant services in line with usual care at the participating sites. The newly developed weekend allied health service allowed managers at each site to reprioritise tasks being performed and the balance of hours provided by each professional group and on which days they were provided. Analyses conducted on an intention-to-treat basis demonstrated that there was no estimated effect size difference between groups in the proportion of patients staying longer than expected (weekend versus no weekend; estimated effect size difference [95% CI], *p*-value) in Trial 1 (0.40 versus 0.38; estimated effect size difference 0.01 [−0.01 to 0.04], *p =* 0.31, CI was both above and below non-inferiority margin), but the proportion staying longer than expected was greater with the newly developed service compared to its no weekend service control condition (0.39 versus 0.40; estimated effect size difference 0.02 [0.01 to 0.04], *p =* 0.04, CI was completely below non-inferiority margin) in Trial 2. Trial 1 and 2 findings were discordant for the mean length of stay outcome (Trial 1: 5.5 versus 6.3 days; estimated effect size difference 1.3 days [0.9 to 1.8], *p <* 0.001, CI was both above and below non-inferiority margin; Trial 2: 5.9 versus 5.0 days; estimated effect size difference −1.6 days [−2.0 to −1.1], *p <* 0.001, CI was completely below non-inferiority margin). There was no difference between conditions for the proportion who had an unplanned readmission within 28 days in either trial (Trial 1: 0.01 [−0.01 to 0.03], *p =* 0.18, CI was both above and below non-inferiority margin; Trial 2: −0.01 [−0.02 to 0.01], *p =* 0.62, CI completely below non-inferiority margin). There was no difference between conditions in the proportion of patients who experienced any adverse event in Trial 1 (0.01 [−0.01 to 0.03], *p =* 0.33, CI was both above and below non-inferiority margin), but a lower proportion of patients had an adverse event in Trial 2 when exposed to the no weekend allied health condition (−0.03 [−0.05 to −0.004], *p =* 0.02, CI completely below non-inferiority margin). Limitations of this research were that 1 of the trial wards was closed by the healthcare provider after Trial 1 and could not be included in Trial 2, and that both withdrawing the current weekend allied health service model and installing a new one may have led to an accommodation period for staff to adapt to the new service settings. Stepped-wedge trials are potentially susceptible to bias from naturally occurring change over time at the service level; however, this was adjusted for in our analyses.

**Conclusions:**

In Trial 1, criteria to say that the no weekend allied health condition was non-inferior to current weekend allied health condition were not met, while neither the no weekend nor current weekend allied health condition demonstrated superiority. In Trial 2, the no weekend allied health condition was non-inferior to the newly developed weekend allied health condition across all primary outcomes, and superior for the outcomes proportion of patients staying longer than expected, proportion experiencing any adverse event, and mean length of stay.

**Trial registration:**

Australian New Zealand Clinical Trials Registry ACTRN12613001231730 and ACTRN12613001361796

## Abstract 2

In two cluster randomised controlled trials in Australia, Terry Haines and colleagues assess the effectiveness of allied health services, such as physical therapy and social work, provided on the weekends in acute medical and surgical hospital wards.

## Author summary

**Why was this study done?:**

- This study was done to find out whether a health service that is routinely provided has the effect that it is intended to have.
- It considers whether it is worth continuing to provide this service when the resources we use to support this service could potentially be better used elsewhere in the health system.

**What did the researchers do and find?:**

- We conducted 2 large trials across 6 acute medical and surgical wards in each of 2 hospitals.
- We removed the weekend allied health (e.g., physical therapy, occupational therapy, and social work) service in the first trial, redesigned it, and reintroduced it in the second trial.
- We found that removing the weekend allied health service had an uncertain impact on patient flow (the change in the percentage of patients who stayed longer than expected was +1% [95% CI: −1% to 4%]) and safety (the change in the percentage with any adverse event was +1% [95% CI: −1% to 3%]), but that having no weekend allied health service on these wards was no worse than having the newly developed service that was brought back in, and indeed was superior for some outcomes (the percentage who stayed longer than expected was 2% better [95% CI: 1% to 5%], and the percentage with any adverse event was 3% better [95% CI: 0% to 5%] with no weekend service).

**What do these findings mean?:**

- These findings indicate that resources spent on providing allied health services on weekends across acute medical and surgical wards could possibly be better spent elsewhere in the healthcare system.
- This study also demonstrated that it is possible to undertake research of routinely provided services where it is uncertain whether they provide the benefits they are intended to.

## Introduction

Disinvestment is the process of (partially or completely) withdrawing health resources from any existing healthcare practices, procedures, technologies, or pharmaceuticals that are deemed to deliver little or no health gain for their cost and thus are not efficient health resource allocations [1]. Governments and professional bodies around the world are introducing processes to eliminate or limit access to services and procedures known to deliver little or no health gain for their cost. Examples from the United States include the Choosing Wisely campaign driven by the American Board of Internal Medicine Foundation [2], and the Patients Before Paperwork initiative led by the American College of Physicians [3].

There is some controversy surrounding what the key motivation for these disinvestments should be. Commentators have expressed concern that the Choosing Wisely campaign is seen by some as a cost-cutting exercise, and feel that it would be more acceptable if the focus were placed on helping to avoid patient harm that may arise from unnecessary tests and ineffective interventions [2]. We would argue that disinvesting from ineffective services could be seen as being more virtuous and acceptable if entwined with an argument based on opportunity costs: that resources spent delivering ineffective services to one patient are the same resources that can no longer be used to deliver an effective service to improve health outcomes for another. This position is best seen from the perspective of healthcare administrators with finite budgets who must choose between competing services they can offer with their available funds.

Much of the debate thus far has centred on disinvestment from individual services that are known to be ineffective or unnecessary, particularly for certain patient subgroups. But what is to be done with classes of services that are routinely provided, but have unknown effectiveness or cost-effectiveness? Continued provision of these services, if ineffective, creates an opportunity cost that is wasteful and stops other patients from potentially benefitting from those same resources. If services are effective, cessation of provision creates a situation for potential patient harm and poorer health outcomes than what otherwise would have been achieved. Thus, decision makers are left in an unenviable position—by virtue of the absence of evidence relevant to guide their decision—of choosing between continued provision, cessation, or reduction, which each carry an element of risk for their patients. This situation is further complicated in circumstances where a service is provided by clinicians who believe it is effective, despite the absence of evidence to this effect. Planning to remove such a service may create fear of negative patient and/or provider outcomes in staff members, which can act as a barrier to practice change [4,5].

One such area of uncertainty is the delivery of allied health services on weekends to patients in acute medical and surgical units. Allied health services (such as physical therapy or social work) are now commonly provided on weekends in hospitals internationally, though not to the same extent as during the week [6–10]. These models appear to have increased in popularity following observational studies identifying a ‘weekend effect’—poorer patient and service outcomes associated with admissions or procedures taking place on weekends [11–15]. However, the efficacy of these services has not been established. A recent, methodologically inclusive systematic review of these services amongst acute joint arthroplasty patients found some benefit in improving length of stay and function [16]. However, this finding was largely driven by findings of observational studies, and no randomised trials examining the efficacy of weekend allied health services in acute settings were identified. Links between allied health staffing levels and the weekend effect have not been established, and some have questioned the very existence of the weekend effect [17,18].

The aim of this study was to establish the impact of disinvesting from provision of allied health services on weekends across acute medical and surgical hospital wards.

## Methods

Human Research Ethics Committee approval for this project was provided by the Monash Health (approval ref 13327B) and Melbourne Health (approval number: 2013.283) Human Research Ethics Committees.

### Design

**Fig 1:**
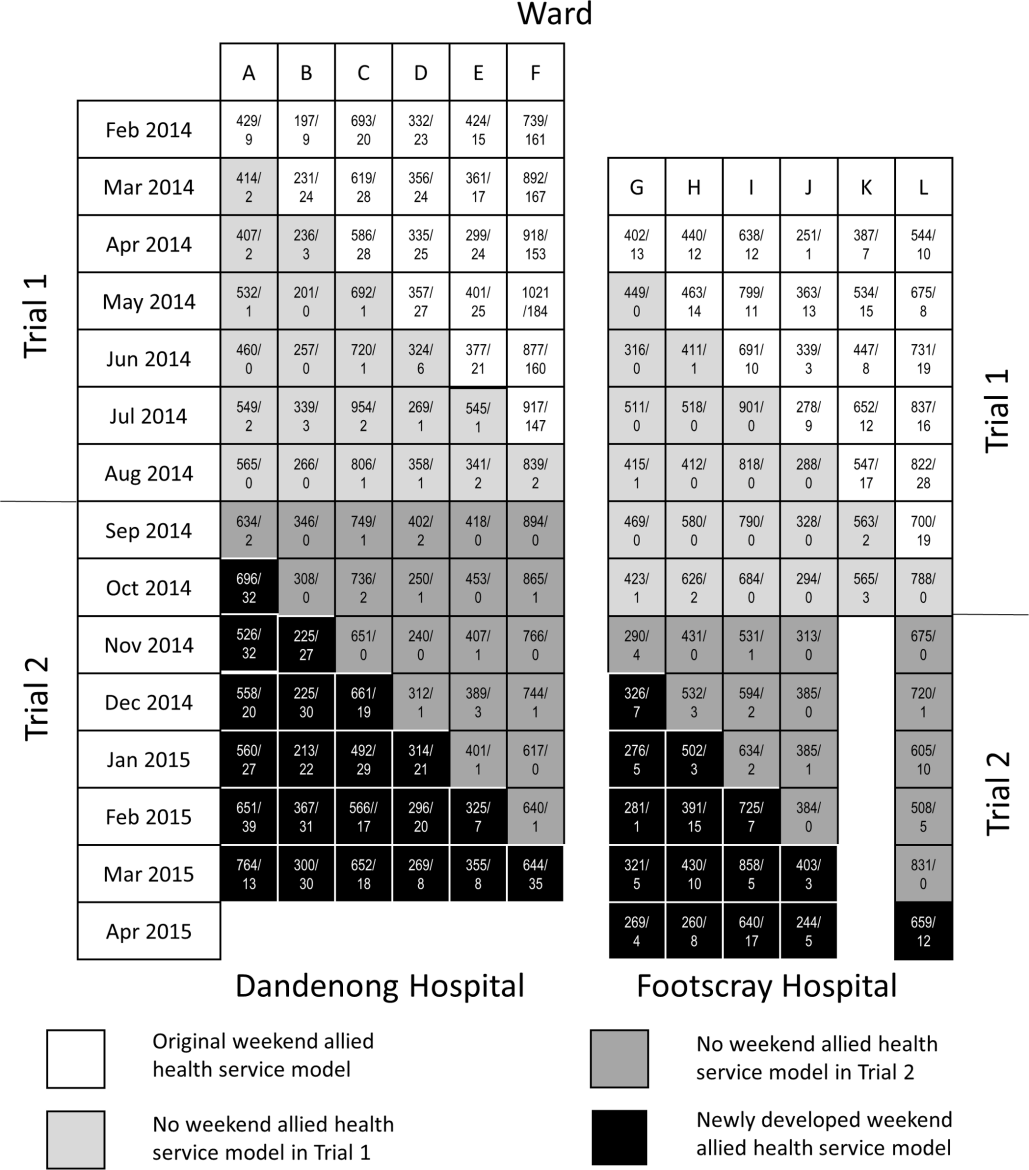
Trial design and occasions of allied health service delivery on weekdays and weekends within each month of the trial. Numbers in boxes indicate occasions of service provided by allied health team on weekdays/on weekends that month. These data include approved clinical exceptions during no weekend service periods.

We have previously proposed an approach for use in the context of disinvestment from a service that has unknown effectiveness [4]. This approach is centred on simultaneous disinvestment from the health service in question while also generating the evidence examining the effectiveness of this service. In this research, we conducted 2 stepped-wedge cluster randomised controlled trials across each of 2 tertiary, metropolitan teaching hospitals, Dandenong Hospital and Footscray Hospital, in Victoria, Australia. Stepped-wedge trials are a form of cluster randomised trial with unidirectional crossover that are increasingly being used for health service evaluations [19]. Trial 1 was a disinvestment randomised trial where the ‘current’ weekend allied health service was removed from 1 ward per calendar month in an order determined at random. It contained examination of both non-inferiority and superiority hypotheses for each primary outcome examined, and superiority hypotheses for each secondary outcome examined. Testing non-inferiority hypotheses does not preclude testing of superiority hypotheses and can be done without statistical penalty [20]. Trial 2 was a conventional stepped-wedge randomised trial where a ‘newly developed’ weekend allied health service was reintroduced in a 180-degree rotated image of Trial 1 (Fig 1) [5]. This reintroduction of weekend allied health services in Trial 2 was considered necessary in the present context given the incremental and evolving development of the current weekend service delivery models that had taken place at the participating sites combined with heterogeneity in delivery models of these services internationally. The newly developed model that was reintroduced was intended to be a complex intervention that used a reproducible procedure for development. Its development involved gathering feedback from medical, nursing, and allied health staff working on the targeted wards. This feedback was reviewed by and discussed with local allied health managers in charge, who made the final decision as to the make-up of the newly developed model.

There were 3 deviations from the analysis plan and 1 change to the outcome measures described in our study protocol (S1 Text). First, a pre-planned meta-analysis that combined data from both sites and trials was not performed due to discordance in findings between Trial 1 and Trial 2 [21]. Second, a sensitivity analysis for Trial 1 was performed where a 1-month analysis washout period was introduced immediately following introduction of the ‘no weekend allied health service’ condition: the September 2014 block at Dandenong Hospital and the November 2014 block at Footscray Hospital served as the last block for this sensitivity analysis of Trial 1. Third, analyses without adjustment for monthly outcome data for the previous 2 years were undertaken. These analysis changes were inspired by peer-reviewers. The secondary outcome of staff absenteeism could not be extracted in clinical units relevant for this trial, so was abandoned. A CONSORT checklist for this study is provided as S2 Text.

### Participants and setting

This research took place across 6 acute medical or surgical wards at Dandenong Hospital and 6 at Footscray Hospital, Victoria, Australia. Most of these wards were specialised in different areas of medical/surgical medicine and admitted different types of patients. At Dandenong Hospital, the ward specialties were orthopaedic surgery, stroke, thoracic/vascular/general surgery & medical, general medicine, head/neck/plastics, and surgical. At Footscray Hospital, the ward specialties were medical (2 wards), infectious diseases/respiratory, plastics/ENT/head/neck surgery, general surgery/colorectal/breast/endocrine/urology, and general surgery/vascular/thoracic/upper gastrointestinal. Patient allocation to wards was driven by patient specialty requirements and bed availability.

Patients who were exposed to the no weekend allied health service condition as well as either the current or newly developed weekend allied health service conditions were excluded to avoid research-design-induced contamination. The requirement to collect individual patient-level consent for researchers to access the primary and secondary outcomes reported in this paper was waived during the ethical approval process.

### Randomisation and masking

Random allocation of wards at each site to starting position in the trial design was undertaken at public meetings at each site. Investigators (D. Mitchell and EHS) developed a list of pseudonyms to represent each ward and then provided these to another investigator (TPH) who was blinded to the meaning of each pseudonym. This investigator then used a random number generator command in Microsoft Excel to allocate wards to starting positions in the stepped-wedge trial design. From this point, staff and patients were not blinded to group allocations due to the practical need to notify staff when the weekend allied health service on their ward would cease.

### Interventions

A detailed description of weekend allied health service models investigated in both of these trials (including TIDiER checklist [22]) and the process used to develop the newly designed model in Trial 2 was provided in our protocol [5]. Briefly, we undertook extensive consultation with relevant stakeholders (medical, nursing, and allied health staff and managers) on participating wards. These staff were interviewed (group interviews at the ward level and individual/key informant interviews) by investigators experienced in conducting qualitative and participatory action research (LO, FM). These staff were not asked to say which professional discipline they wanted to be employed on the weekends, rather, to identify and prioritise the tasks that they believe to be most important for allied health to perform on weekends in terms of improving patient health outcomes, improving patient flow, and reducing readmissions. They were also asked to reflect on the strengths and limitations of the current model of care, suggest areas for improvement, and examine patient incident and clinical exception data gathered during Trial 1 to inform their decisions. Allied health managers were provided with this list of tasks and other feedback gathered, so they could propose the new stakeholder-driven model of weekend allied health service they felt would work best. A Delphi meeting was used to facilitate this process separately at each hospital site [23]. This information was forwarded to each hospital’s allied health director, who made the final decision.

Services delivered under the original (Trial 1) and newly developed models (Trial 2) at each site are presented in S3 and S4 Texts, which present the amount of service provided by each discipline group when the service was fully operational (first month of Trial 1 and last month of Trial 2).

The no weekend service condition entailed delivery of no allied health services on weekends to wards affected, unless criteria for a clinical exception had been met. A safety mechanism specified in our trial protocol was that a process be established whereby the trial protocol could be violated for an individual patient if specific, pre-planned criteria set by local clinicians and approved by local administrators were met (S5 Text).

### Outcomes

The primary outcomes were indicators of the domains of patient flow through the hospital (length of stay measured in days, proportion of patients staying longer than their diagnosis-related group average inlier length of stay), failures in discharge planning (proportion of patients with unplanned readmissions within 28 days of discharge), and failures in patient care (proportion of patients experiencing any of the following adverse events: in-hospital fall, Code Blue call, Medical Emergency Team call, pulmonary embolus, deep vein thrombosis, death, hospital-acquired pressure area, or intensive care unit admission from the ward). We used 2 indicators of patient flow due to the inherent limitations of using the intuitively attractive indicator (length of stay measured in days) in the context of our stepped-wedge design. The results of a stepped-wedge trial can be biased if certain hospital wards, but not others in the same trial, change the types of patients (particularly those with greater or lesser lengths of stay) they tend to admit over time to cope with seasonal demands. Statistically adjusting for patient diagnosis and procedure type is a possible approach to dealing with this problem, but only if the number of meaningful diagnostic/procedural groupings is small enough to enable statistical models to be validly calculated (which is not the case in this context). Our alternate approach was to use the dichotomous outcome of whether each patient stayed longer than expected for their diagnosis/procedural-related grouping. In Australia, the Australian Refined Diagnosis Related Groups approach is employed. Hospital data coders classify patients into these groupings that are based on similarity of conditions and usage of hospital resources, using information in the hospital morbidity record such as the diagnoses, procedures, and demographic characteristics of the patient [24]. The primary outcomes were collected through hospital administrative data systems and checked daily by research assistants interviewing ward representatives and checking handover documentation.

Secondary outcomes collected across all participants included the proportion of patients discharged to residential aged care facilities, the cost (in Australian dollars) per patient to the healthcare system per admission, the proportion of patients discharged on a Saturday or Sunday, and the number of compliments and complaints (total and allied health specific). These outcomes were extracted from hospital administrative data systems at completion of the study. The cost of inpatient treatment per patient was extracted from hospital-based clinical costing systems in August 2016 to allow finalisation of hospital costing processes. It should be noted here that clinical costing data are largely driven by length of stay, and hence this outcome has the same limitations as described above for the length of stay outcome.

Process measures collected included the occasions of weekend allied health service delivery (i.e., the number of times an allied health professional went and saw a patient on the weekend) and reason for clinical exceptions taking place. Occasions of allied health service delivery were recorded by allied health staff, collected through routine hospital administrative data systems, and extracted at the end of the study. The frequency and reason for clinical exceptions taking place were recorded by site investigators (KM, TC, and MS) who had local responsibility for approving these at the time of the exception being granted. Non-patient-related outcomes and qualitative, economic, subsample, and meta-regression analyses described in our protocol were reserved for further publications.

### Procedure

An audit was conducted prior to the trial to establish the average amount of weekend service per ward and the financial costs of the overall service. During Trial 1, weekend services were titrated down according to pre-trial audit amounts. If a ward received 4 hours of physical therapy over a weekend prior to trial commencement, it is this amount that was removed when this service was ceased on this ward. For Trial 2, the service was reintroduced on a pro rata basis. So, if a hospital provided A$2,400 in allied health staffing costs per weekend across 6 wards overall prior to the trial, then A$400 worth of resources per ward was allowed to be reintroduced when the newly developed service was commenced. At the Dandenong Hospital site, managers reallocated their funds to increase the overall number of allied health hours provided by moving some hours to a Friday afternoon and Monday morning. This was intended to facilitate discharge planning prior to the weekend and immediately following and to maximise the total number of allied health hours. At the Footscray Hospital site, managers changed the personnel who provided the weekend allied health service from casual staff to allied health staff working in the intensive care unit and the Immediate Response Service, whose usual responsibility was to screen and assess presentations to the emergency department to prevent inappropriate admissions and facilitate discharge planning.

**Fig 2:**
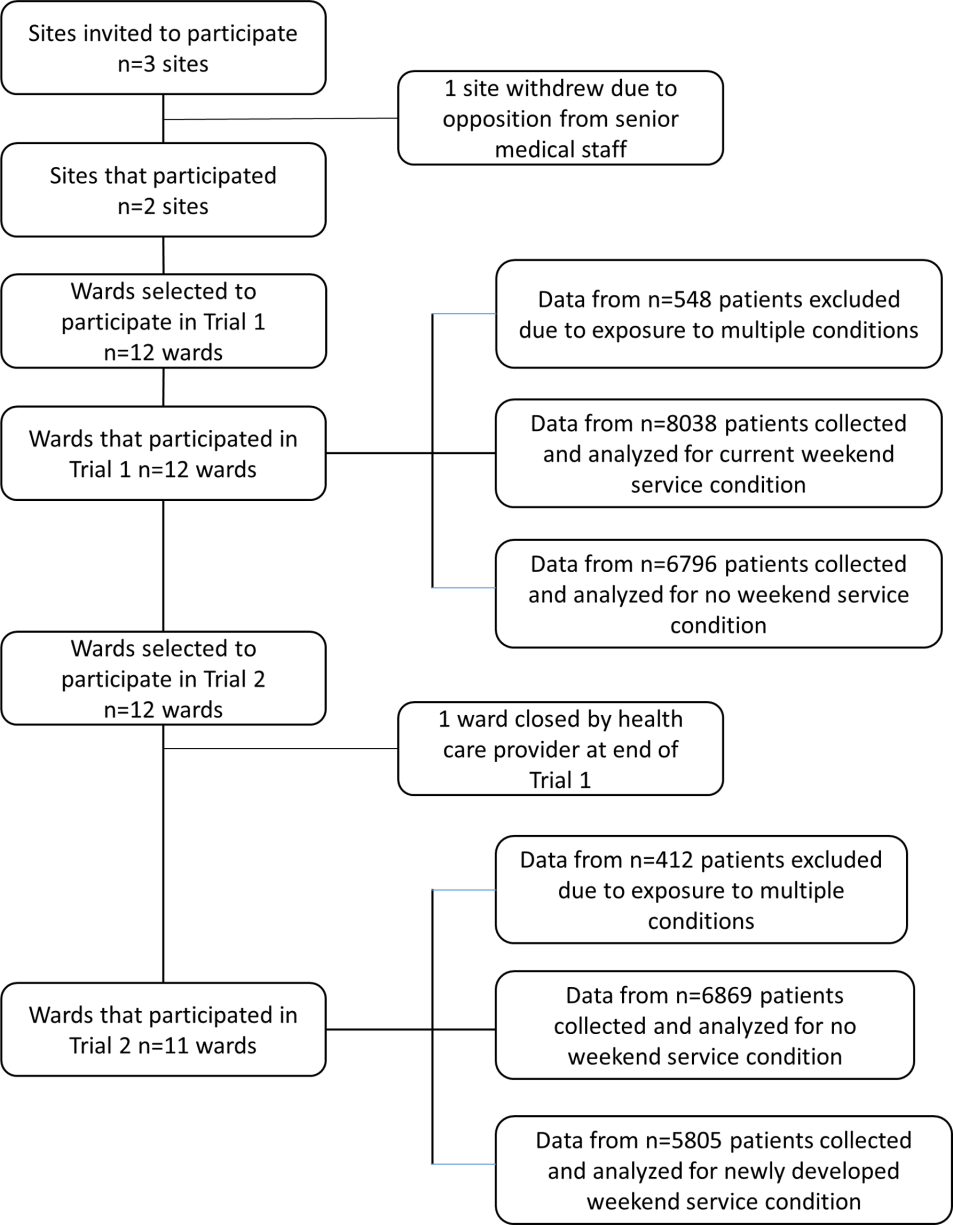
CONSORT flow chart of site, ward, and patient involvement in data collection and analysis.

A CONSORT flow chart (Fig 2) provides study recruitment, data collection, and analysis details. Nursing staff managers on each ward were engaged prior to study commencement to identify which tasks usually performed by allied health staff on weekends could be modified, accelerated (to a Friday), delayed (to a Monday), or transferred to other staff who were present on weekends. They were also provided with the criteria for clinical exceptions on their wards. Study stopping rules and non-inferiority margins were also developed prior to trial commencement by hospital administrators and allied health managers at participating sites [5].

Project research assistant data collectors were present 7 days per week during the study period. They collected data through medical record review and interviews with hospital staff, enabling crosschecking of data collected between routine hospital administrative data systems and direct data collection approaches [25,26].

These trials were registered with the Australian New Zealand Clinical Trials Registry. Trial 1 was prospectively registered on 8 November 2013 (ACTRN12613001231730), and Trial 2 on 12 December 2013 (ACTRN12613001361796).

### Statistical analysis

Multilevel, mixed-effects generalised linear model analyses were used to generate effect size estimates and 2-tailed 95% CIs and superiority hypothesis *p*-values. The 95% CIs were compared to the non-inferiority margins for each primary outcome measure to determine if they were completely below (indicating non-inferiority), completely above (indicating inferiority), or both above and below (indicating uncertain inferiority) the non-inferiority margin. The non-inferiority margins were an absolute difference of 2% in proportion outcomes (proportion of patients staying longer than the average inlier diagnosis-group-related length of stay, proportion with an unplanned readmission within 28 days, proportion experiencing 1 or more of the adverse events previously listed) and an absolute difference of 1 day for the mean length of stay outcome.

These analyses were conducted by a statistician (SM) independent to the research locations and blinded to ward allocation status through use of 6 mock codes representing different ward allocation patterns. An intention-to-treat analysis approach was employed. Models were initially constructed using patient-level data nested within ward nested within hospital site. Patient admissions were coded against the first study ward of their admission and calendar month of their first admission to a study ward. Log natural transformation of length of stay data was planned due to the anticipated skewed distribution [5]. Effect estimates were generated using ward-month-level data if effect estimates could not be calculated using patient-level data (on account of inability to ascertain starting values for models with binary outcomes). All analyses were adjusted for study month and ward as categorical fixed factors in line with recommendations for analysis of stepped-wedge designs [19]. Adjustment was also made for monthly outcome data from the previous 2 years for primary outcomes to account for potential seasonal fluctuations that are consistent from year to year. The main effect of having no weekend service was calculated, followed by pre-planned ‘no weekend service by site’ interaction effects to determine if site-level analyses were required [27]. Analyses were undertaken using Stata MP v.14 (StataCorp, College Station, Texas).

Three post hoc exploratory analyses were undertaken. The first investigated potential differences in outcomes between the 2 weekend allied health service delivery models (current versus newly developed) directly. This analysis was considered important as there was no guarantee that the newly developed model would be superior to the current model despite this being the intent of redesigning the weekend allied health service. The second examined whether the results under the no weekend service condition were comparable between the 2 trials. Previous studies have reported substantial changes over time within health services in outcomes examined in this trial (e.g., falls [28]), making it important to consider whether the no weekend allied health service conditions in the 2 trials were comparable. Adjustment for study month was not used in these exploratory analyses as this would have led to collinearity with the independent variables of interest. The third post hoc analysis was a sensitivity analysis for Trail 1 whereby a 1-month washout period was applied to each ward following their transition to the no weekend allied health service condition. This meant that the month of data immediately following the transition in model of care was excluded from the analysis for each ward and that the final month included in the analysis of Trial 1 became September 2014 for the Dandenong Hospital site and November 2014 for the Footscray Hospital site. This analysis was important as changes in practice can potentially affect hospital practices and processes in a way that is different to what happens once the change has had more time to become imbedded in practice.

Data were monitored by a committee drawn from senior clinical staff at participating sites who were not members of the study investigative team. Trial dates are provided (Fig 1), along with deviations from the registered trial protocol (Fig 2; inability to commence at 1 site and closure of 1 ward during Trial 2). Study data used in these analyses are provided (S1 and S2 Data). Study power analysis calculations are provided (S6 Text) and were described in our protocol paper [5].

## Results

Trial dates of commencement and completion along with occasions of allied health service are presented (Fig 1). Demographic characteristics of trial participants (Trial 1, *n =* 14,834; Trial 2, *n =* 12,674) are presented (Table 1). There were 28 approved clinical exceptions in total (14 in each trial) during the no weekend allied health periods, of which the most common justification was a post-fall mobility review by a physiotherapist (16). The remaining occasions of service provision were not approved clinical exceptions during this period.

**Table 1:** Participant demographics for each group within each trial.

Characteristic	Trial 1		Trial 2	
	Current weekend service	No weekend service	No weekend service	Newly developed weekend service
*N*	8,038	6,796	6,869	5,805
Age (years)—mean (SD)	59.5 (20.7)	60.8 (20.2)	59.7 (20.6)	59.8 (20.3)
Sex—*n* (%) male	4,225 (53.6%)	3,611 (53.1%)	3,587 (52.2%)	3,090 (53.2%)
Most common Australian Refined Diagnosis Related Groups—*n* (%)	Other digestive system disorders, 267 (3.3%); respiratory infections and inflammations, 214 (2.6%); heart failure and shock, 211 (2.6%); chronic obstructive airways disease, 208 (2.6%)	Chronic obstructive airways disease, 250 (3.7%); respiratory infections and inflammations, 248 (3.7%); other digestive system disorders, 202 (3.0%); septicaemia, 192 (2.8%)	Other digestive system disorders, 237 (3.5%); septicaemia, 198 (2.9%); chronic obstructive airways disease, 179 (2.6%); respiratory infections and inflammations, 178 (2.6%)	Other digestive system disorders, 194 (3.3%); chronic obstructive airways disease, 162 (2.8%); respiratory infections and inflammations, 153 (2.6%); laprascopic cholecystectomy, 145 (2.4%)
Expected length of stay (days)*—mean (SD)	5.3 (5.1)	5.7 (5.1)	5.0 (4.5)	5.4 (5.6)

*Based on mean inlier Australian Refined Diagnosis Related Groups from Victoria in 2013.

Data for primary and secondary outcomes for the full trial sample are presented (Table 2). Effect size estimates for each trial are presented (Table 3). The *p*-values presented in Table 3 reflect the probability of the findings observed when the superiority null hypothesis is true, and were derived from multilevel, mixed-effects generalised linear model analyses. Length of stay was greater for the no weekend service condition in Trial 1, but shorter in Trial 2. The no weekend allied health condition was of uncertain inferiority in Trial 1 for this outcome (95% CI: 0.85 to 1.77), but non-inferior in Trial 2 (95% CI: −2.03 to −1.13) relative to the +1 day non-inferiority margin. The proportion of patients staying longer than their diagnosis-related group average inlier length of stay was lower for the no weekend service condition in Trial 2 compared to the newly developed weekend allied health service condition. The no weekend allied health service condition was of uncertain inferiority for the proportion of patients who stayed longer than expected (95% CI: −0.01 to 0.04), the proportion who had an unplanned readmission within 28 days (95% CI: −0.01 to 0.03), and the proportion who had 1 or more adverse events (95% CI: −0.01 to 0.03) in Trial 1 relative to a +2% absolute increase non-inferiority margin. However, the no weekend allied health service condition was non-inferior to the newly developed weekend allied health service condition for the proportion of patients who stayed longer than expected (95% CI: −0.05 to −0.01), the proportion who had an unplanned readmission within 28 days (95% CI: −0.02 to 0.01), and the proportion who had 1 or more adverse events (95% CI: −0.05 to −0.004). It was also superior for the proportion of patients staying longer than expected and the proportion who had 1 or more adverse event outcomes.

**Table 2:** Raw data for primary and secondary outcomes.

Outcome	Trial 1		Trial 2	
	Current weekend service	No weekend service	No weekend service	Newly developed weekend service
**Length of stay—mean (SD)/median (IQR)**	5.5 (7.5)/3.1 (1.5 to 6.4)	6.3 (9.4)/3.7 (1.7 to 7.2)	5.0 (5.9)/3.2 (1.6 to 6.1)	5.9 (8.3)/3.4 (1.6 to 6.9)
**Patients staying longer than expected—*n* (%)**	3,263 (40.2%)	2,608 (38.4%)	2767 (40.3%)	2288 (39.4%)
**Patients with an unplanned readmission within 28 days—*n* (%)**	788 (9.8%)	733 (10.8%)	671 (9.8%)	577 (9.9%)
**Adverse events**				
Any adverse event—*n* (%)	685 (8.5%)	665 (9.8%)	608 (8.9%)	506 (8.7%)
Fall—*n* people (%)/*n* events	165 (2.1%)/212	140 (2.1%)/181	156 (2.3%)/218	88 (1.5%)/105
Code Blue/MET call—*n* people (%)/*n* events	300 (3.7%)/400	323 (4.7%)/461	263 (3.8%)/345	274 (4.7%)/380
Pulmonary embolus—*n* people (%)/*n* events	17 (0.2%)/17	11 (0.2%)/11	10 (0.2%)/10	4 (0.1%)/5
Deep vein thrombosis—*n* people (%)/*n* events	8 (0.1%)/8	7 (0.1%)/8	8 (0.1%)/8	4 (0.1%)/5
Death—*n* (%)	143 (1.8%)	171 (2.5%)	135 (2.0%)	120 (2.1%)
Pressure area—*n* people (%)/*n* events	80 (1.0%)/99	72 (1.1%)/78	58 (0.8%)/61	26 (0.5%)/28
ICU admission from the ward—*n* people (%)/*n* events	154 (1.9%)/167	172 (2.5%)/197	146 (2.1%)/157	153 (2.6%)/173
**Discharge destinations**				
Home/private residence—*n* (%)	6,566 (81.7%)	5,468 (80.5%)	5,367 (78.1%)	2,758 (82.0%)
Aged care facility—*n* (%)	142 (1.8%)	159 (2.3%)	171 (2.5%)	137 (2.4%)
Other acute/extended care/rehab hospital—*n* (%)	693 (8.6%)	579 (8.5%)	663 (9.7%)	451 (7.8%)
Cost per patient to the healthcare system per admission*—mean (SD)/median (IQR)	8,446 (11,205)/5,095 (2,688 to 10,168)	9,501 (13,661)/5,550 (2,988 to 10,796)	8,233 (9,942)/5,223 (2,797 to 100,062)	9,442 (14,794)/5,334 (3,001 to 10,153)
**Compliments**				
Total	32^£^	33^€^	38^£^	24^€^
Allied health specific	1^£^	3^€^	2^£^	0^€^
**Complaints**				
Total	37^£^	32^€^	33^£^	43^€^
Allied health specific	0^£^	0^€^	1^£^	1^€^

*Cost in Australian dollars.

^£^Data from first month of trial only.

^€^Data from last month of trial only.

ICU, intensive care unit; MET, Medical Emergency Team.

**Table 3:** Effect size estimates of main and trial-by-site interaction effects from each trial for primary and secondary outcomes.

Outcome	Trial 1				Trial 2			
	Main effect	Inferiority	Intervention-by-site interaction	ICC*	Main effect	Inferiority	Intervention-by-site interaction	ICC*
**Primary**								
Length of stay (days)	1.31 (0.85 to 1.77) *p <* 0.001^£^	Uncertain inferiority	0.03 (−0.57 to 0.63) *p =* 0.93	S: 0.0001 W: 0.04 E: 0.91	−1.59 (−2.03 to −1.13) *p <* 0.001^£^	Non-inferior	0.23 (−0.42 to 0.88) *p =* 0.48	S: <0.0001 W: 0.03 E: 0.88
Length of stay (log transformed)	0.09 (0.04 to 0.15) *p =* 0.002	N/A	−0.07 (−0.15 to 0.01) *p =* 0.08	S: <0.0001 W: 0.10 E: 0.91	−0.14 (−0.21 to −0.08) *p <* 0.001	N/A	−0.03 (−0.13 to 0.06) *p =* 0.47	S: 0.02 W: 0.08 E: 0.89
Proportion of patients staying longer than expected	0.01 (−0.01 to 0.04) *p =* 0.30	Uncertain inferiority	−0.02 (−0.06 to 0.02) *p =* 0.34	S: 0.83 W: 0.86	−0.02 (−0.05 to −0.01) *p =* 0.04^£^	Non-inferior	−0.02 (−0.07 to 0.02) *p =* 0.30	S: 0.85 W: 0.88
Proportion with an unplanned readmission within 28 days	0.01 (−0.01 to 0.03) *p =* 0.18	Uncertain inferiority	−0.04 (−0.06 to −0.02) *p <* 0.001^£^	S: <0.0001 W: 0.39	−0.01 (−0.02 to 0.01) *p =* 0.62	Non-inferior	−0.02 (−0.05 to 0.01) *p =* 0.18	S: <0.0001 W: 0.53
Proportion of patients with any adverse event	0.01 (−0.01 to 0.03) *p =* 0.33	Uncertain inferiority	−0.02 (−0.04 to 0.01) *p =* 0.15	S: <0.0001 W: 0.71	−0.03 (−0.05 to −0.004) *p =* 0.02^£^	Non-inferior	0.02 (−0.01 to 0.05) *p =* 0.24	S: <0.0001 W: 0.70
**Secondary**								
Proportion of patients discharged to aged care facility	0.001 (−0.004 to 0.01) *p =* 0.28	N/A	−0.003 (−0.01 to 0.01) *p =* 0.54	S: 0.30 W: 0.65	−0.001 (−0.01 to 0.01) *p =* 0.88	N/A	0.008 (−0.01 to 0.01) *p =* 0.99	S: 0.12 W: 0.62
Cost to the healthcare system per admission (Australian dollars)	1,810 (1,094 to 2,525) *p <* 0.001	N/A	769 (−168 to 1,706) *p =* 0.11	S: <0.0001 W: 0.013 E: 0.76	−2,431 (−3,166 to −1,696) *p <* 0.001^£^	N/A	−163 (−1,273 to 947) *p =* 0.77	S: <0.0001 W: 0.005 E: 0.88
Proportion of patients discharged on a Saturday or Sunday	0.01 (−0.01 to 0.03) *p =* 0.33	N/A	0.02 (−0.01 to 0.05) *p =* 0.24	S: <0.0001 W: 0.43	−0.02 (−0.05 to −0.002) *p =* 0.03^£^	N/A	−0.01 (−0.04 to 0.02) *p =* 0.69	S: <0.0001 W: 0.47

Main effects are interpreted as the impact of being exposed to the ‘no weekend’ allied health condition compared to the ‘current’ or ‘newly developed’ weekend allied health conditions. Data in parentheses are 95% CIs.

*Intraclass correlation coefficients (ICCs) derived from mixed-effects generalised linear models partitioned at the site (S), ward (W), and patient episode (E) levels.

^£^Statistically significant (superiority hypothesis, 2-tailed *p <* 0.05).

N/A, not applicable.

Examination of site-by-intervention interaction effects identified an interaction for the proportion of patients with an unplanned readmission in Trial 1. The subsequent subgroup analyses indicated that at the Dandenong Hospital site the no weekend service condition had a higher proportion of unplanned readmissions (coefficient [95% CI]: 0.03 [0.01 to 0.05], *p =* 0.01), but this was not the case at the Footscray Hospital site (coefficient [95% CI]: −0.0002 [−0.03 to 0.02], *p =* 0.86).

Examination of superiority hypotheses for secondary outcomes demonstrated that there was difference between conditions in the proportion of patients discharged to residential aged care facilities. However, the cost to the healthcare system per admission was greater for the no weekend service condition in Trial 1, but less in Trial 2.

Examination of superiority hypotheses for the secondary outcome of the proportion of patients discharged on a Saturday or Sunday demonstrated that there was no difference between conditions in Trial 1, but proportion discharged on a Saturday or Sunday was marginally (2%) higher for the newly developed weekend service in Trial 2. This finding should be viewed in the context that length of stay was longer when a weekend allied health service was present in that trial.

Exploratory analyses (Table 4) indicated that patients in the no weekend service condition in Trial 1 had better outcomes across multiple domains than those in the no weekend service condition in Trial 2. Patients exposed to the original weekend allied health service delivery model did not experience different outcomes than those exposed to the newly developed model for the primary outcomes of the proportion staying in hospital longer than expected, the proportion who had an unplanned readmission within 28 days, and the proportion experiencing any adverse event when these patient groups were compared directly. There was a difference for mean length of stay (log transformed) and total cost favouring the original weekend allied health service delivery model, though these outcomes did not account for differences in patient diagnosis categories between phases. The sensitivity analyses of Trial 1, with the washout period added, demonstrated that the no weekend allied health condition was non-inferior to the current weekend allied health condition (95% CI: 0.01 to 0.60) for the length of stay outcome, whereas there was uncertain inferiority for this outcome in the primary analysis. The other outcomes were largely unaffected in this sensitivity analysis.

**Table 4:** Effect size estimates of main effects from the exploratory analyses.

Outcome	Weekend service: ‘current’ versus ‘newly developed’		No weekend service: Trial 1 versus Trial 2		Sensitivity: Trial 1 with 1-month washout period		
	Effect size	ICC*	Effect size	ICC*	Effect size	Inferiority	ICC*
**Primary**							
Length of stay (days)	0.12 (−0.20 to 0.43) *p =* 0.46	S: 0.0002 W: 0.06 E: 0.89	1.03 (0.73 to 1.32) *p <* 0.001^£^	S: <0.0001 W: 0.01 E: 0.91	0.30 (0.01 to 0.60) *p =* 0.04^£^	Non-inferior	S: <0.0001 W: 0.05 E: 0.91
Length of stay (log transformed)	0.05 (0.01 to 0.09) *p =* 0.02^€^	S: 0.0001 W: 0.12 E: 0.90	0.11 (0.07 to 0.15) *p <* 0.001^£^	S: 0.0001 W: 0.06 E: 0.90	0.05 (0.02 to 0.09) *p =* 0.005^£^	N/A	S: <0.0001 W: 0.01 E: 0.91
Proportion of patients staying longer than expected	0.01 (−0.01 to 0.04) *p =* 0.31	S: 0.83 W: 0.86	0.01 (−0.003 to 0.03) *p =* 0.004^£^	S: 0.87 W: 0.89	0.03 (−0.004 to 0.06) *p =* 0.09	Uncertain inferiority	S: 0.83 W: 0.87
Unplanned readmission within 28 days	0.01 (−0.01 to 0.02) *p =* 0.42	S: <0.0001 W: 0.45	−0.001 (−0.02 to 0.01) *p =* 0.95	S: <0.0001 W: 0.50	0.01 (−0.01 to 0.03) *p =* 0.31	Uncertain inferiority	S: <0.0001 W: 0.39
Proportion of patients with any adverse event	−0.002 (−0.01 to 0.01) *p =* 0.74	S: 0.02 W: 0.68	0.02 (0.008 to 0.04) *p =* 0.02^£^	S: <0.0001 W: 0.59	0.01 (−0.01 to 0.03) *p =* 0.33	Uncertain inferiority	S: <0.0001 W: 0.71
**Secondary**							
Proportion of patients discharged to aged care	−0.004 (−0.01 to 0.003) *p =* 0.30	S: 0.14 W: 0.58	−0.01 (−0.01 to 0.003) *p =* 0.09	S: 0.23 W: 0.63	−0.001 (−0.01 to 0.01) *p =* 0.89	N/A	S: 0.31 W: 0.70
Cost to the healthcare system per admission (Australian dollars)	−558 (−1,086 to −30) *p =* 0.04^£^	S: 0.0001 W: 0.02 E: 0.86	1,224 (745 to 1,702) *p <* 0.001^£^	S: 0.0001 W: 0.01 E: 0.76	952 (494 to 1,410) *p <* 0.001^£^	N/A	S: 0.0001 W: 0.03 E: 0.79
Proportion of patients discharged on a Saturday or Sunday	−0.01 (−0.04 to 0.02) *p =* 0.34	S: <0.0001 W: 0.43	0.05 (−0.01 to 0.10) *p =* 0.10	S: <0.0001 W: 0.47	0.02 (−0.004 to 0.04) *p =* 0.12	N/A	S: <0.0001 W: 0.51

Main effects are interpreted as the impact of being exposed to the ‘current’ service, exposure to Trial 1, and exposure to the ‘current’ service for the three analyses, respectively. Data in parentheses are 95% CIs.

*Intraclass correlation coefficients (ICCs) derived from mixed-effects generalised linear models partitioned at the site (S), ward (W), and patient episode (E) levels.

^£^Statistically significant (superiority hypothesis, 2-tailed *p <* 0.05).

N/A, not applicable.

Analyses without adjustment for monthly outcome data for the previous 2 years had some inconsistencies with main analyses, where the adjustment was made (S7 Text). Differences in the proportion of patients staying longer than expected and with any adverse event were no longer significant between the no weekend service and newly developed weekend allied health service conditions in Trial 2. However, the 95% CIs were still completely below the non-inferiority margin, meaning that the interpretation of these results from this perspective remains unaffected. There were also site-by-intervention interaction effects for the log-transformed length of stay (Trial 1) and proportion of patients with any adverse event (Trial 2) outcomes. Here, the log-transformed mean length of stay was longer if exposed to the no weekend allied health service condition at the Dandenong Hospital (coefficient [95% CI]: −0.15 [0.07 to 0.23], *p <* 0.001), and the proportion of patients who experienced any adverse event was lower in the no weekend allied health service condition at Dandenong Hospital only (coefficient [95% CI]: −0.03 [−0.05 to −0.002], *p =* 0.03).

## Discussion

In Trial 1, criteria to say that the no weekend allied health condition was non-inferior to current weekend allied health condition were not met, while neither the no weekend nor current weekend allied health condition demonstrated superiority. The result for the mean length of stay outcome from Trial 1 was sensitive to whether a 1-month washout period was applied in the analysis. When the washout period was applied, the no weekend allied health service condition was found to be non-inferior to the current weekend allied health service model. In Trial 2, the no weekend allied health condition was non-inferior to the newly developed weekend allied health condition across all primary outcomes, and superior for the proportion of patients staying longer than expected, proportion experiencing any adverse event, and mean length of stay.

The findings of this study were somewhat discordant with the only previously published randomised trial of the effect of weekend allied health services on patient and health service outcomes. This earlier trial reported no significant difference in length of stay or patient adverse events (*p* > 0.05) in those provided with a Saturday physical therapy service, in addition to a Monday to Friday service, on rehabilitation wards [29]. However, this trial did identify small benefits of the service in terms of improved functional independence and health-related quality of life attributable to the intervention, though these benefits were arguably below clinically meaningful thresholds [30–32]. It is difficult to directly compare these results given the differences in ward types and patient populations involved, the fact that the earlier study focused only on a physical therapy service, and the differences in the activities undertaken by allied health staff between acute and rehabilitation settings. Our study was the first to our knowledge to use this particular disinvestment research design to simultaneously disinvest from a routinely provided service with uncertain effectiveness while also developing evidence that had previously been missing as to the effectiveness of the intervention.

It should be noted that the models of care we examined in Trial 1 were limited to those in place at the outset of the trial at the study locations. However, the model of care in Trial 2 was a complex intervention, where the process used could be reproduced in other settings to develop models that are similarly tailored to local conditions. A strength of these two trials in addressing the research context area was their size, which led to narrow confidence intervals in our analyses and a high degree of certainty in the results. Our choice to investigate both the current weekend allied health service and a newly developed service also enhanced the generalisability of our findings to real world settings. However, these trials did not investigate the breadth of all possible permutations and combinations of weekend allied health service delivery models. Rather, we focused on the pragmatic scenario of what was currently being allocated and the budgetary envelope of this. Our study was limited in that we were unable to proceed with this research on 1 ward at 1 of our sites in Trial 2 due to closure of that clinical unit. We were also unable to proceed at a third site due to local opposition to participation in the study. This highlights some of the difficulties that can be encountered in conducting disinvestment research of this nature over an extended period of time. This study could have been further strengthened by considering the healthcare costs consumed by patients after they were discharged from these acute wards. It is possible that allied health services not provided on weekends in the acute setting may still have been provided in another setting later in a patient’s journey.

The scope of the clinical implications of our findings should be clearly defined. The study locations did not have all types of specialty wards (e.g., spinal, burns) that might be considered to be acute medical or surgical wards. The finding of no effect of weekend allied health services overall should not be extrapolated to weekday services. It is possible that variation in staffing profile and the lack of availability of community-based services on weekends that support patients upon discharge are an important difference. We also did not withhold weekend allied health services from those who met our clinical exception criteria. The low frequency of these exceptions may indicate that using staff from other areas that do have a weekend allied health service (e.g., intensive care unit) or using an on-call staffing model may be preferable to employing weekend allied health staff in fixed shifts to meet these patients’ needs.

This study reported discordant findings for the mean length of stay and total cost outcomes between Trials 1 and 2. This can be explained by 5 potential mechanisms. First, these changes could be attributed to background variation in patient case mix not accounted for by these outcomes. Winter months in Australia, which largely coincided with the no weekend services condition in Trial 1, are associated with a 20% increase in demand for medical admissions primarily due to infectious diseases [33]. Second, it may be that the current weekend allied health service delivery model was superior to the newly developed model. Our exploratory analyses directly comparing these models did not support this explanation as the log-transformed length of stay outcome favoured the newly developed service when these conditions were directly compared. Third, the current service model could be argued to have had an unfair advantage in a direct comparison with the newly developed service model. The current service model had several years of refinement locally and integration into usual care before being subjected to this evaluation, whereas the newly developed service model was evaluated as soon as it was introduced and did not have the same opportunity to be refined and integrated. Fourth, the patient cohort or standard of usual care provided at study sites could have changed within the no weekend service periods across Trials 1 and 2. Our exploratory analyses identified multiple outcomes where differences favoured those exposed to the no weekend service condition during Trial 2 compared to Trial 1, which would support this hypothesis. The fifth potentially concurrent explanation is that both withdrawing the weekend allied health service model and installing a new one required an accommodation period for staff to adapt to the new service settings. This hypothesis would also be supported by the differences observed between Trials 1 and 2 within the no weekend service periods. Models of organisational lag have been formally investigated since the 1980s [34] and may indicate that our original research design should have included a washout period following the transition to the new model of care. Our sensitivity analyses, where we introduced a 1-time-period washout to Trial 1, identified that the length of stay in the no weekend service condition was non-inferior to the current weekend service condition (though other results were not substantially changed).

There is potential that naturally occurring change over time could confound the results of these stepped-wedge trials. Naturally occurring change over time (maturation) is a potential confounder in every longitudinal interventional research design. The important concern is whether this may have biased the intervention effect size estimates calculated from each trial. In a stepped-wedge design, this problem is prominent given the unidirectional crossover employed. This means that a stepped-wedge trial conducted within a system/organisation that naturally improves over time will favour the condition that is tested second, and vice versa. This problem was first described in detail by Hussey and Hughes [35]. Their solution to this problem was to explicitly model the effect of each time period to eliminate this bias from the intervention effect estimate. We used this approach in every analysis presented in Table 3. Thus, our estimated effect sizes calculated from within Trial 1 and Trial 2 are statistically independent of the potentially confounding effects of change over time that occurred across the organisations involved, and can be considered to be free of bias from this source. The analyses that were not independent of these effects are the comparisons between Trial 1 and Trial 2 that are reported in Table 4. We could not use this approach in the comparisons between the 2 control periods, and in the direct comparisons of the current and the newly developed weekend services in Table 4 as these were not comparisons based on a stepped-wedge design. Rather, these were pre- versus post-intervention design comparisons with no overlap in time periods between the conditions being compared (thus a time covariate would directly confound the covariate of interest, which in this case was Trial 1 versus Trial 2). The washout sensitivity analysis model in Table 4 did use the approach described by Hussey and Hughes as this was a stepped-wedge design.

The key implication of this research is that resources being used to support weekend allied health service delivery to acute medical and surgical wards similar to those involved in this study could potentially be put to better use elsewhere in the healthcare system. Future research in the field of weekend allied health service delivery is warranted, particularly in other ward types and when examining higher dosage levels of service delivery. If higher dosage levels can demonstrate an effect on clinical or patient flow outcomes, they will still need to be justified economically. Future research using this disinvestment trial approach is also warranted. The value of a trial that finds a model of care that no longer includes a particular service to be non-inferior to one that includes it can be conceptualised as the future opportunity costs saved of no longer providing that service. Hence, commonly provided services that have a relative absence of evidence supporting their use and a high opportunity cost of delivery should be targeted.

## Supporting information

S1 Data Ward-level data. (XLSX)

S2 Data Patient-level data. (XLSX)

S3 Data Blinded statistician data. (XLSX)

S1 Text Trial protocol. Study protocol for 2 randomised controlled trials examining the effectiveness and safety of current weekend allied health services and a new stakeholder-driven model of weekend allied health services for acute medical/surgical patients versus no weekend allied health services.
(PDF)

S2 Text CONSORT checklist. (PDF)

S3 Text TIDieR criteria. (DOCX)

S4 Text Hours of service by discipline. Hours of allied health service provision offered under current and newly developed weekend allied health service delivery models at each site.
(DOCX)

S5 Text Clinical exceptions. (DOCX)

S6 Text Power analysis. (DOCX)

S7 Text Analyses unadjusted for monthly ward data from previous 2 years. Effect size estimates of main and trial-by-site interaction effects from each trial for primary and secondary outcomes. Main effects are interpreted as the impact of being exposed to the no weekend allied health condition compared to the current or newly developed weekend allied health conditions.
(DOCX)
