# Supplementary material for: Impact of disinvestment from weekend allied health services across acute medical and surgical wards: 2 stepped-wedge cluster randomised controlled trials
Source: PLoS Med. 2017 Oct 31;14(10):e1002412. doi: 10.1371/journal.pmed.1002412 (PMC5663333; doi:10.1371/journal.pmed.1002412)
Supplement: S1 Text — Study protocol for 2 randomised controlled trials examining the effectiveness and safety of current weekend allied health services and a new stakeholder-driven model of weekend allied health services for acute medical/surgical patients versus no weekend allied health services. (PDF) [file pmed.1002412.s004.pdf]

Haines et al. Trials (2015) 16:133
DOI 10.1186/s13063-015-0619-z

TRIALS

STUDY PROTOCOL Open Access

Study protocol for two randomized controlled trials examining the effectiveness and safety of current weekend allied health services and a new stakeholder-driven model for acute medical/surgical patients versus no weekend allied health services

Terry P Haines[1*], Lisa O'Brien[1,2], Deb Mitchell[1,3], Kelly-Ann Bowles[1], Romi Haas[1], Donna Markham[3], Samantha Plumb[4], Timothy Chiu[5], Kerry May[3], Kathleen Philip[6], David Lescai[6], Fiona McDermott[7], Mitchell Sarkies[3], Marcelle Ghaly[5], Leonie Shaw[4], Genevieve Juj[4] and Elizabeth H Skinner[1,5]

Abstract

Background: Disinvestment from inefficient or ineffective health services is a growing priority for health care systems. Provision of allied health services over the weekend is now commonplace despite a relative paucity of evidence supporting their provision. The relatively high cost of providing this service combined with the paucity of evidence supporting its provision makes this a potential candidate for disinvestment so that resources consumed can be used in other areas.

This study aims to determine the effectiveness, cost-effectiveness and safety of the current model of weekend allied health service and a new stakeholder-driven model of weekend allied health service delivery on acute medical and surgical wards compared to having no weekend allied health service.

Methods/Design: Two stepped wedge, cluster randomised trials of weekend allied health services will be conducted in six acute medical/surgical wards across two public metropolitan hospitals in Melbourne (Australia). Wards have been chosen to participate by management teams at each hospital. The allied health services to be investigated will include physiotherapy, occupational therapy, speech therapy, dietetics, social work and allied health assistants. At baseline, all wards will be receiving weekend allied health services. Study 1 intervention will be the sequential disinvestment (roll-in) of the current weekend allied health service model from each participating ward in monthly intervals and study 2 will be the roll-out of a new stakeholder-driven model of weekend allied health service delivery. The order in which weekend allied health services will be rolled in and out amongst participating wards will be determined randomly. This trial will be conducted in each of the two participating hospitals at a different time interval. Primary outcomes will be length of stay, rate of unplanned hospital readmission within 28 days and rate of adverse events. Secondary outcomes will be number of complaints and compliments, staff absenteeism, and patient discharge destination, satisfaction, and functional independence at discharge.

(Continued on next page)

* Correspondence: Terrence.Haines@monash.edu
[1]Allied Health Research Unit, Monash Health and Physiotherapy Department, Monash University, Melbourne, Victoria, Australia
Full list of author information is available at the end of the article

BioMed Central

© 2015 Haines et al.; licensee BioMed Central. This is an Open Access article distributed under the terms of the Creative Commons Attribution License (http://creativecommons.org/licenses/by/4.0), which permits unrestricted use, distribution, and reproduction in any medium, provided the original work is properly credited. The Creative Commons Public Domain Dedication waiver (http://creativecommons.org/publicdomain/zero/1.0/) applies to the data made available in this article, unless otherwise stated.

(Continued from previous page)

Discussion: This is the world's first application of the recently described non-inferiority (roll-in) stepped wedge trial design, and the largest investigation of the effectiveness of weekend allied health services on acute medical surgical wards to date.

Trial registration: Australian New Zealand Clinical Trials Registry.
Registration number: ACTRN12613001231730 (first study) and ACTRN12613001361796 (second study).
Was this trial prospectively registered?: Yes.
Date registered: 8 November 2013 (first study), 12 December 2013 (second study).
Anticipated completion: June 2015.
Protocol version: 1.
Role of trial sponsor: KP and DL are directly employed by one of the trial sponsors, their roles were: KP assisted with overall development of research design and assisted with overall project management; DL contributed to project management, administration and communications strategy.

Keywords: Disinvestment, Hospital, Effectiveness, Randomised trial, Allied health

Background

Allied health services (such as physiotherapy, occupational therapy, speech pathology, social work and dietetics) are now commonly provided on the weekend in hospitals internationally. A survey of tertiary-care hospitals in Canada reported that 97% of facilities provided weekend physiotherapy services [1]. This was at a lower intensity than during the week and there was high variability in the scope of services provided between hospitals. In an Australian study, 61% of hospitals provided physiotherapy on Saturdays, and 45% on Sundays [2]. There is little published information examining weekend services amongst other allied health disciplines. One survey of Australian public hospital emergency departments found that 6 out of 21 responding hospitals had rostered occupational therapy weekend services [3].

There is a body of indirect evidence indicating that provision of earlier and higher intensities of particular allied health services improves health outcomes for a range of hospital patient populations [4-8]. However, increasing service provision on weekends may not have the same effect as increasing services during the week. Staff who are employed in weekend roles may not have the same level of organisational knowledge/expertise/connection as staff who work during the week, which may affect patient health outcomes, discharge planning and subsequent organisational flow outcomes. Also, community support services that allied health services may refer patients to may not operate over the weekend, reducing their ability to facilitate discharges at this time.

There is scant evidence from well-designed studies that directly supports the effectiveness of allied health services provided over the weekend. A systematic review of experimental, quasi-experimental and observational studies concluded that research to date did not provide strong evidence that physiotherapy services provided on the weekend reduced length of stay, improved patient discharge mobility status or discharge destination [9]. Subsequent research has focused on provision of physiotherapy ± occupational therapy on weekends in rehabilitation wards and has generated some support for service effectiveness in this context [10-13]. However, patients on rehabilitation wards are distinct from those in other hospital wards given their longer length of stay and higher need for rehabilitation therapy. Hence, it is difficult to extrapolate the findings of research conducted in rehabilitation settings to other hospital areas such as acute medical/surgical wards.

An additional factor that should influence whether allied health services are provided on weekends is that of economic efficiency. There are two key factors that may limit the cost-effectiveness of allied health services provided over the weekend, being: the higher cost per hour of employing staff over weekends compared to during the week, [14] and the possibility of diminishing marginal returns [15]. This latter principle suggests that the amount of additional benefit gained for each additional unit of service provision will decrease as the overall level of service provision increases, meaning that the cost-effectiveness ratio of these services will decrease with increasing levels of service provision (that is, there may be less benefit achieved from increasing allied health services from 5 to 7 days service per week than when increasing from 3 to 5 days of service per week). There is some evidence from an observation dose-response study indicating this principle applies to physiotherapy rehabilitation services [16].

Direct evidence of the economic efficiency of allied health services delivered on weekends is scant and inconsistent in its support of weekend allied health services. A partial economic evaluation (cost evaluation) of a weekend physiotherapy service provided to rheumatology patients in the United Kingdom found increased costs of service provision with no reduction in length of

stay (indeed, a non-significant increase of 0.5 days) associated with this programme [17]. A quasi-experimental, historical control group study found weekend physiotherapy provided to patients who had undergone a total hip or knee arthroplasty generated a cost saving to the health fund driven by a reduction in length of stay from 12.28 days to 10.84 days [18]. An observational study of a 7-day a week, 24-hour, on-call social work service provided in a hospital's emergency department suggested that the programme was operated at little cost to the hospital [19]. However, this study was purely an accounting exercise based on the estimated accounts paid and actual cost of the on-call services. An economic evaluation arising from a randomised controlled trial investigating the cost-effectiveness of additional Saturday physiotherapy and occupational therapy services on rehabilitation inpatients compared to those receiving usual Monday to Friday services had an incremental cost utility ratio of AUD 41,825 (95% confidence interval (CI) −2,817 to 74,620) per quality-adjusted life year (QALY) gained for the intervention group indicating this approach in this setting is likely to be cost-saving [20].

Clearly, there is uncertainty as to whether providing weekend allied health services is effective or cost-effective, particularly when provided on acute medical or surgical wards. Current widespread provision of these services complicates conduct of a traditional randomised trial as the intervention is already being provided as a part of usual care (the default control condition in pragmatic research). Our research team has recently devised a novel disinvestment research design that can be applied in the context where a health technology is being applied as a part of routine care, yet there is uncertainty as to the effectiveness, cost-effectiveness or safety of this health technology [21]. In this research, we will use this novel research approach to evaluate the effectiveness, cost-effectiveness and safety of two models of weekend allied health service being provided on acute medical and surgical wards compared to having no weekend allied health service.

Methods/Design

Design

This research comprises two studies. Study 1 will consist of two hospital sites undertaking a novel, stepped wedge, roll-in, cluster randomised disinvestment trial whereby the current model of weekend allied health service delivery will be 'rolled back in' (withdrawn). Study 2 will consist of these same two hospital sites undertaking a conventional stepped wedge, roll-out, cluster randomised trial design in which a new stakeholder-driven model of weekend allied health service delivery will be rolled out to the same wards. Study 2 will commence immediately following completion of study 1.

Participants, therapists, centres

This research will take place across six acute medical or surgical wards from Dandenong Hospital and Western Hospital (Footscray), in Victoria, Australia. Both of these hospitals are major tertiary, metropolitan hospitals. The wards (Table 1) were selected by project investigators in consultation with managers and clinicians based at each site on the basis of currently having a weekend allied health service, the patient types being treated on that ward (medical or surgical patients, not rehabilitation), and not being anticipated to undergo major structural change (for example, substantive change of patient case-mix or refurbishment requiring ward shut-down) during the study period which would confound the trial design. High-risk wards such as intensive and coronary care units, emergency departments and paediatric wards were excluded. Each hospital will commence this research at

Table 1 Caseload in participating wards

Hospital	Ward	Description
Dandenong Hospital	SW3/W2 acute	Orthopaedic surgery
	SW4	Stroke unit
	West 3	Thoracic, Vascular, General surgical and medical units
	West 4	General medicine
	North Ward	Head and neck, Plastics
	North 3	Surgical
Western Hospital	2B	Medical
	2C	Medical
	2D	Infectious diseases; Respiratory medicine
	2 W[a]	Plastics; Head and neck surgery; ENT surgery
	3 W	General surgery; Colorectal; Breast; Endocrine; Urology
	3E	General surgery; Vascular surgery; Thoracic; Upper gastrointestinal

[a]Study 1 only.

different time points (Dandenong Hospital commencing February 2014 and Western Hospital (Footscray) commencing April 2014.

Data in this study will be collected from three groups of participants. Each group of participants is now described in detail.

Group 1

'All patients on participating wards.' This group will consist of patients admitted to acute medical or surgical wards involved in the trial who are over the age of 18 years. Paediatric patients will be excluded from this study as they are not routinely seen on the selected wards (none are paediatric units or have paediatric beds) and have different provisions for service delivery. It is anticipated, based on previous patient flow data, that each ward will have an average of 174 patient admissions per month during the trial. This means that 6 wards at 1 site will have 7,308 patient admissions during the 7 months of study 1 and 7,308 during the 7 months of study 2. This will lead to an anticipated total of 29,232 patient admissions during the overall study period.

Group 2

'Randomly selected subgroup of patients from participating wards'. A randomly selected subgroup of approximately 600 patients from participating wards will be recruited for additional data collection. Study data collectors will use a random number generator to randomly select wards on specific days during the study period that they will attend and approach all patients who are planned for discharge within the next 24 hours from that ward to consent to participate in this component of the study. These participants will be recruited to contribute data to secondary outcomes for this study.

Group 3

'Health professionals.' A volunteer subgroup of medical, nursing and allied health staff (four to twelve per participating ward) will be sought to participate in qualitative data collection approaches (group interviews and key informant interviews) being used as a part of the project process evaluation and planning for the intervention model to be used in study 2. This will include staff who work on the participating wards during the week and on the weekend. Three waves of data collection will take place with these health professionals (pre-study 1, between studies 1 and 2, and post-study 2).

Intervention/Control

We have provided a summary of our intervention conditions described according to the TIDieR guidelines [22] in Table 2. Further elaboration of the intervention conditions is now provided according to study.

Study 1 intervention condition - current weekend allied health services

Current physiotherapy, occupational therapy, social work, dietetics, speech pathology and allied health assistant weekend service delivery on the participating wards will be the intervention for study 1 of this trial. During this study, the weekend allied health services of each participating ward (cluster) will be 'rolled in' sequentially using the stepped wedge design (Figure 1 and Additional file 1). The current model of weekend allied health service delivery in the hospitals participating in this study has not been developed systematically, rather in an *'ad hoc'* manner driven by decisions made by individual allied health managers. Thus, there is potential that this model is not currently tailored to the needs of individual wards and is, therefore, not delivering optimal outcomes. However, it is the prevailing model of care that in a pragmatic research design serves as an appropriate reference standard [23].

Design at Western Hospital (Footscray) is similar but with 5 wards involved in study 2 and a 13-month total study period. Black = current weekend allied health service. White = no weekend allied health service. Grey = new, stakeholder-driven weekend allied health service. Twelve months of pre-trial data collection at participating sites indicated that weekend allied health services at each site varied in terms of the number of disciplines involved, the amount of time allocated per ward and the total budgetary allocation to this activity. Steps will be taken during study 1 to ensure intervention fidelity relative to the pre-trial data collection. Allied health staff providing weekend services will be updated as to the amount of time spent on each ward per month and will have pre-trial data used as monthly targets to ensure that service provision levels within study 1 intervention periods are consistent with pre-trial data. Staff will be advised to continue performing tasks and prioritising patients in the same manner with which they had been doing so prior to trial commencement.

Study 1 - control condition - no weekend allied health services

The control condition will be characterised by the provision of no weekend allied health services unless a patient meets a 'clinical exception' criteria. A 'clinical exception' refers to circumstances where the trial treatment protocol is permitted to be violated due to the risk of harm to an individual or organisation. This is also intended to mitigate against possible protocol violations by staff and to ease their concerns regarding the health and well-being of patients given the novel research

Table 2 Intervention conditions according to TIDieR criteria

TIDieR criteria	Study 1 intervention	Study 2intervention
Item 1. Brief name: provide the name or a phrase that describes the intervention	Usual care weekend allied health service	Stakeholder-driven weekend allied health service
Item 2. Why: describe any rationale, theory, or goal of the elements essential to the intervention	Usual care is the prevailing model of care in the research location that in a pragmatic research design serves as an appropriate reference standard. This model of care has developed incrementally over time and has largely been driven by decisions of individual allied health managers in an *'ad hoc'* manner	A new model of weekend allied health service will be developed where managers and staff of participating wards are engaged to identify the most important tasks that require completion on weekends that could be undertaken by allied health staff. It is anticipated that by directly engaging with these key stakeholders, a new model of care that better meets the needs of individual wards will be developed
Item 3. What (materials)?: describe any physical or informational materials used in the intervention, including those provided to participants or used in intervention delivery or in training of intervention providers	There are no specific materials used beyond those materials ordinarily used by allied health professionals during the week. It is left to the discretion of individual staff what materials they use in their clinical practice	There are no specific materials used beyond those materials ordinarily used by allied health professionals during the week. If the people involved in developing this model of care determine that additional materials are required, these will be identified and described at a later date
Item 4. What (procedures)?: describe each of the procedures, activities, and/or processes used in the intervention, including any enabling or support activities	Allied health services may include services provided by physiotherapy, occupational therapy, social work, dietetics, speech pathology professionals and allied health assistants. Services delivered are the same as those performed on weekdays, although the intensity of weekend services is lower (fewer hours per ward) than weekday services. Services commonly include mobilisation, chest physiotherapy, discharge planning, assessment and prescription of aids and equipment, swallowing assessment, dietary analysis and prescription, and counselling	Services provided are likely to be similar to that of the usual care weekend allied health service. However, the people involved in developing this model will be able to inform the practitioners involved of the relative priority of the different tasks that they may be asked to perform on each ward
Item 5. Who provided?: for each category of intervention provider (for example, psychologist, nursing assistant), describe their expertise, background and any specific training given	All allied health professionals will have entry-level allied health degrees as a minimum. Orientation of new staff members to the health care organisation and wards that they work on is provided as a part of standard human resources procedures. Allied health assistants do not require formal qualification but most have a certificate III or IV [44] and all operate under the direction of an allied health professional	Services providers are likely to be similar to that of the usual care weekend allied health service. However, the people involved in developing this model will be able to decide which service providers are best positioned to undertake the tasks that require completion on the weekend, and will also decide if transdisciplinary training is required by individual practitioners
Item 6. How?: describe the modes of delivery (such as face to face or by some other mechanism, such as Internet or telephone) of the intervention and whether it was provided individually or in a group	Face to face individual patient interaction	Face to face individual patient interaction
Item 7. Where: describe the type(s) of location(s) where the intervention occurred, including any necessary infrastructure or relevant features	Hospital acute medical/surgical ward environment	Hospital acute medical/surgical ward environment
Item 8. When and how much?: describe the number of times the intervention was delivered and over what period of time including the number of sessions, their schedule, and their duration, intensity or dose	Individual patients will receive variable amounts of weekend allied health service delivery. The intensity of services provided is at the discretion of the allied health professional. The number of hours of weekend allied health service delivered per day will vary between wards within each site and between sites	Individual patients will receive variable amounts of weekend allied health service delivery. The intensity of services provided is at the discretion of the allied health professional. The number of hours of weekend allied health service delivered per day will vary between wards within each site and between sites
Item 9. Tailoring: if the intervention was planned to be personalised, titrated or adapted, then describe what, why, when, and how	All weekend allied health services will be tailored to the needs of the patients being treated. This will be at the discretion of the treating allied health professional based upon their clinical judgement.	All weekend allied health services will be tailored to the needs of the patients being treated. This will be at the discretion of the treating allied health professional based upon their clinical judgement.
Item 10. Modifications: if the intervention was modified during the course of the study,	Not applicable for protocol	Not applicable for protocol

Table 2 Intervention conditions according to TIDieR criteria *(Continued)*

describe the changes (what, why, when, and how)		
Item 11. How well (planned)?: if intervention adherence or fidelity was assessed, describe how and by whom, and if any strategies were used to maintain or improve fidelity, describe them	Research assistants will be present daily on study wards to both promote and monitor intervention fidelity in terms of which wards should be receiving weekend allied health services and which should not. Patient contact statistics are recorded by allied health professionals and are recorded in hospital administrative datasets. These datasets will be used by investigators to measure time spent by weekend allied health personnel with patients on each ward.	Research assistants will be present daily on study wards to both promote and monitor intervention fidelity in terms of which wards should be receiving weekend allied health services and which should not. Patient contact statistics are recorded by allied health professionals and are recorded in hospital administrative datasets. These datasets will be used by investigators to measure time spent by weekend allied health personnel with patients on each ward.
Item 12. How well (actual)?: If intervention adherence or fidelity was assessed, describe the extent to which the intervention was delivered as planned	Not applicable for protocol	Not applicable for protocol

approach being used in this study [21]. Patients who meet one of these criteria will be permitted to see a weekend allied health staff member despite them being on a ward during a 'control' period. Local nursing or medical staff will identify if the patient meets the predetermined set of criteria, which allows for them to be seen by an allied health professional over a weekend. This will be escalated to the site allied health director or project site liaison who will approve provision of the service following a final check against the 'clinical exception' criteria. These criteria will be suggested by ward-level staff during pre-trial group interviews, and managers through key-informant and group interviews and will vary from site to site. Staff will be asked to provide evidence to support their request for a clinical exception criteria (for example, research papers, local incident reports, hospital policy), and project investigators will determine whether the strength of evidence presented warrants formation of a clinical exception criterion.

The frequency of clinical exceptions under each criterion will be recorded to monitor control condition fidelity. Project data collectors at each site will be present on

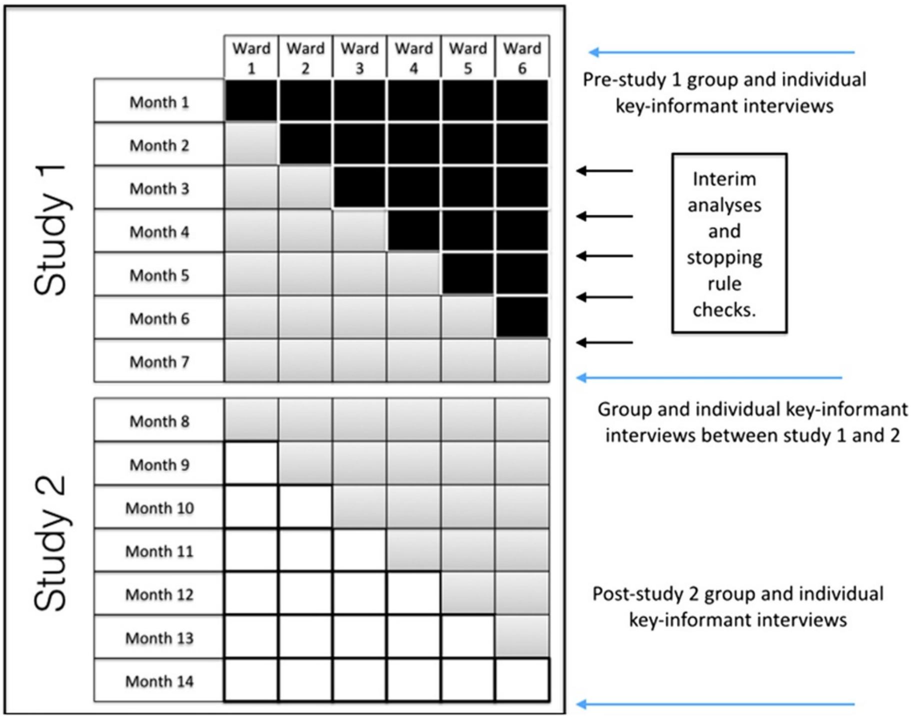

Figure 1 **Design of study 1 and study 2 at Dandenong Hospital.**

each ward each day of this project and will facilitate ongoing communication with ward staff regarding local clinical exception criteria.

Study 2 - control condition - no weekend allied health services

This will be identical to the study 1 control condition.

Study 2 - intervention condition - stakeholder-driven model of weekend allied health services

A criticism of using the current model of weekend allied health service provision as the intervention in study 1 is that it is possible that the sites involved may have constructed their prevailing model on an *ad hoc* basis, which may not reflect higher standards of service set in other hospitals. This is consistent with evidence already discussed that there is high variability in the amount and focus of allied health services already being delivered on weekends internationally [1]. However, there is no existent 'gold standard' for how a weekend allied health service delivery model should be structured (which services, how much, what activities) given the paucity of evidence regarding the comparative efficacy of different weekend allied health service delivery models. Developing such a standard across all medical and surgical wards may also be an impossibility, as different types of wards will have different patient casemix and different allied health service requirements.

Rather than conform to a non-existent 'standard' for what a weekend allied health service delivery model should look like, we have sought to standardise a process by which an 'optimal' site-specific service could be developed. Thus, the model of allied health service delivery used as the intervention in study 2 of this research is a complex intervention [24].

Our process for developing this site-specific weekend allied health service delivery model begins with extensive consultation with relevant stakeholders (medical, nursing and allied health staff and managers) on participating wards. These staff will be interviewed (group and individual, key informant) to drive development of this model; hence, we refer to this model as a stakeholder-driven model. Investigators experienced in conducting qualitative and participatory action research (LO, FM) will facilitate these interviews. These staff will not be asked to say which professional discipline they want to be employed on the weekends, rather, to identify and prioritise the tasks that they believe to be most important for allied health to perform on weekends both in terms of improving patient health outcomes, improving patient flow, and reducing readmissions. They will also be asked to reflect on the strengths and limitations of the current model of care, suggest areas for improvement and to examine patient incident and clinical exception data gathered during the first study of the trial to inform their decisions. Allied health managers will be provided with this list of tasks and other feedback gathered, so that they can propose what the new, stakeholder-driven model of weekend allied health service delivery will be. Transdisciplinary, interdisciplinary and multidisciplinary models of care are all possible candidates for the model of care that will arise from this process. These managers will then be engaged in a Delphi process [25] to select the preferred model of care from the proposals generated.

Each site will hold constant the budgetary amount allocated to weekend allied health services between studies 1 and 2. During the second 'step' in the stepped wedge design of study 2, the first ward to resume provision of weekend allied health services using the stakeholder-driven model will be provided with a budgetary allocation that is the average per ward of study 1. During the third step, when a second ward resumes provision of weekend allied health services, the total budgetary allocation to be shared between the 2 wards that have resumed provision of the weekend service will be twice the average per ward of study 1. However, provisions within the stakeholder-driven model will be made to allow weekend allied health to distribute their time unequally between the two wards based on the task prioritisation framework provided to them by the stakeholders who designed this model. This provision recognises that an optimal service would target patients who are likely to receive the greatest benefit from this service, regardless of the ward they are physically being treated on.

Outcome measures

Current United Kingdom Medical Research Council guidance on the evaluation of complex interventions indicates that a single primary outcome may not make best use of data in these evaluations, rather, that a range of outcome measures will be needed including possible unintended consequences [23]. In this research, we plan to examine three domains of primary outcome with one domain being analysed in two ways, and another being measured as a composite outcome.

Primary outcome measure: 1) length of stay

Mean overall length of stay per patient who is treated on one of the targeted wards will be used as the primary outcome. This measure is often used as an indicator of hospital efficiency [26]. Length of stay is the key overall driver of inpatient costs and is the most readily available outcome to enable stopping rules to be checked during study 1. However, overall mean length of stay is limited as an outcome for this project as: i) these data can be skewed by highly influential outliers, ii) length of stay of longer-stay patients will not be known until they are

discharged (thus interim analyses for non-inferiority to check stopping rules may underestimate mean length of stay) and iii) patient admission casemix profile may vary over the study period (for example, a study ward may start admitting more patients who typically stay longer). To account for these limitations, we will also examine the proportion of patients who stay longer than their Australian Refined Diagnosis-Related Group average 'inlier' length of stay according to data published from the previous year [27].

Primary outcome measure: 2) rate of unplanned hospital readmission within 28 days

This routinely collected measure will be used as a marker of treatment effectiveness, discharge planning and patient readiness for discharge [28].

Primary outcome measure: 3) rate of adverse events

Patient adverse events will be collected using a range of clinical data collection systems. The events quantified will be in-hospital falls, Code Blue/Medical Emergency Team calls, pulmonary embolus, deep vein thrombosis, death, hospital acquired pressure area, and intensive care unit admission from the ward. Rate of adverse events (events per person-time) will be considered as a single, composite end-point when considered as a primary outcome. A composite end point for this primary outcome domain was selected as a number of different clinical events may indicate a clinical failure, whereas the selection of only one type of clinical event as the end point may not present a comprehensive clinical picture [29].

Secondary outcome measure: 1) number of complaints

Complaints emanating from targeted wards (total and allied health specific) will be captured through the hospital administrative databases and local departmental datasets.

Secondary outcome measure: 2) number of compliments

Compliments emanating from targeted wards will be captured similarly to the number of complaints.

Secondary outcome measure: 3) patient discharge destination

Patient discharge destination will be classified into categories of: i) discharged to the community, ii) transferred to another acute ward, iii) transferred to intensive care, iv) transferred to rehabilitation, and v) discharged to residential aged care.

Secondary outcome measure: 4) patient satisfaction

Patient satisfaction with overall care will be measured using data from the 'overall hospital experience' domain of the Victorian Patient Satisfaction Survey [30]. These data will only be extracted from participant group 2 (randomly selected subgroup of patients from participating wards). These data will not be collected from patients with a diagnosis of dementia or cognitive impairment documented in their medical history.

Secondary outcome measure: 5) patient functional independence at discharge

Patient independence at discharge will be measured using participant self-report on the Modified Barthel Index [31]. These data will be collected from the same participant subgroup as secondary outcome measure: 4) patient satisfaction.

Secondary outcome measure: 6) patient health-related quality of life at discharge

Health-related quality of life at discharge from hospital will be measured using the European Quality of Life, 5 dimensions (EQ-5D-5 L) instrument [32]. These data will be collected from the same participant subgroup as secondary outcome measure: 4) patient satisfaction in study 2.

Secondary outcome measure: 7) staff absenteeism

Staff absenteeism (medical, nursing, allied health staff) will be collected through routine finance reporting. As allied health staff are not allocated to specific wards, but may work across a combination of wards with mixed exposure to intervention and control conditions, we will collect these data at only 3 time points: the first month of study 1, the final month of study 1 and the final month of study 2.

Process measure: 1) allied health hours of service

Number of occasions of allied health service provision to each patient on weekends and weekdays will be collected.

Process measure: 2) clinical exceptions

The frequency and reason for clinical exceptions taking place will be recorded by project research personnel.

Process measure: 3) proportion of patients discharged on a Saturday or Sunday

This outcome is a potential indicator of patient flow during the study. It is important to consider patient flow as a process measure to ensure that bed-block is not occurring on the weekend.

Economic outcome measure: 1) cost of inpatient treatment per patient

Hospital clinical costing data will be used to measure the costs attributed to each patient. If administrative data for a particular patient is unable to be extracted, costing based on the most recent National Weighted Activity Unit calculators [33] will be used. This will be based on the overall

patient admission, not just their time spent on a ward under an intervention or control condition.

Qualitative outcomes
Group and individual interviews will be conducted with staff members from each ward and weekend allied health staff at 3 time points (pre-study 1, between study 1 and study 2, and post-study 2) to explore their satisfaction with and experiences of the different weekend allied health service delivery models being examined.

Procedure
Approval to conduct this study has been obtained from the Monash Health Human Research Ethics Committee (EC00383 - representing Dandenong Hospital) and the Melbourne Health Human Research Ethics Committee (2013.283 - representing Western Hospital (Footscray)) and this trial has been registered with the Australian New Zealand Clinical Trials Registry (ACTRN12613001231730 (study 1) and ACTRN12613001361796 (study 2)). It is anticipated that data collection will commence in February 2014 and be completed in June 2015 with reports and publications finalised by June 2016. A multi-site project executive committee has been developed consisting of all project investigators. This executive committee has developed a project communications plan in consultation with representatives from the Victorian Government, Department of Health. Further pre-trial communications with relevant health services unions, local site clinicians, managers and executives will also be undertaken prior to trial commencement. Extensive staff engagement has been undertaken for this project at both study sites prior to trial commencement.

Randomisation and allocation concealment
The order in which weekend allied health services will be rolled in and out from each participating ward will be determined at random by an investigator blinded to ward identity. One investigator will develop pseudonyms for each ward while another investigator blinded to the true identity of each pseudonym will use a random number generator in Microsoft Excel (Microsoft Corporation, Redmond, WA, USA) to allocate ward number locations to each pseudonym. The first investigator will then be able to reveal which pseudonym represented which ward.

Masking
Research assistants collecting and entering data will not be blinded to the allocation of wards within the stepped wedge research designs. The randomly selected subgroup of patients from participating wards will not be informed of the purpose of the interview as being for the evaluation of a randomised controlled trial. However, we cannot say that all of these participants will be blinded as to the intervention they were exposed to. Masking will be applied to the trial data analyst. Six mock codes representing different orders in which the wards may have progressed through the stepped wedge design (using a Latin Square approach) will be used to blind the statistician conducting the final quantitative analysis from the true identity of each ward and the time sequence in which each ward was randomised.

Trial safety
The process of disinvestment in allied health services has the potential to be detrimental to patient safety and/or organisational outcomes. The checking of stopping rules and development of clinical exceptions will, therefore, be used to ensure safety. A stopping rule allows data to be monitored and the trial to be stopped if there is evidence of a lack of safety or efficacy associated with the intervention [34]. During study 1, interim analyses will be conducted monthly to ensure that patient outcomes have not dropped below a pre-specified non-inferiority margin. This margin represents the maximum amount of gain anticipated if the amount of resources being saved were to be reallocated to another purpose. Discussion groups with clinicians and project investigators initially determined that the non-inferiority margin should be 0.8 multiplied by the standard deviation of the targeted outcome. This is equivalent to 'Cohen's large effect size' [35]. However, review of the standard deviations for the outcomes that these stopping rules would be applied to revealed that these margins were too wide to be acceptable to the management of participating hospitals. As a result of these consultations, the following stopping rules were developed:

1. The 95% CI of the effect (difference between means) of having no weekend allied health services exceeding a 1-day increase in mean length of stay.
2. The 95% CI of the effect (difference between proportions) of having no weekend allied health services exceeding an absolute change of 0.02 (2%) in the outcomes of the proportion of patients who stay longer than their average inlier Australian Refined Diagnosis-Related Group (ARD-RG), the proportion of patients who experience one or more of the previously specified adverse events, or the proportion of patients who are unexpectedly readmitted within 28 days.

Management at each site also reserved the right to review other study data (for example, the proportion of patients discharged on the weekend) in determining whether the study should continue at each site. Interim analysis will be conducted during each month of study 1 and reported back to the project executive committee.

The project executive committee will then forward their recommendations along with blinded data to an independent data-monitoring committee at each site to make a determination as to whether the project should be ceased on the basis of the analysis conducted.

Data will be collected in an identifiable form to allow data linkage with other project datasets but will then be de-identified for storage and analysis once linkages have been made.

Recruitment

A waiver of consent was requested and granted for researchers to access routinely collected, hospital administrative data (for example, length of stay, hospital readmission) covering these forms of data for participants in Group 1. Written consent will be obtained from all participants (where the waiver of consent does not apply). Written consent will be obtained from participants in Group 2 by a project team member (MS, MG) or research assistant. They will be identified as being potentially eligible for consent to participate by screening of ward handover sheets and communication with ward staff. Written consent will be obtained from participants in Group 3 by a project team member (ES, SP, LS, DM, LOB). They will be notified of the possibility of participating in this project by Email and verbal communication.

Data analysis

Sample size estimation

The sample size in this study is governed by the patient throughput on the participating wards over the trial period. Non-inferiority trials do not preclude testing of superiority and can be done so without statistical penalty [36]; hence, we undertook power calculations for studies 1 and 2 from a superiority analysis perspective. Current data from study wards indicates there will be 7,308 patient admissions in total per study per site. We used the approach for conducting power analyses for stepped wedge trials advocated by Hussey and Hughes [37] based upon the Wald statistic. We applied this approach to 3 of our primary outcomes and demonstrated > 90% power in each case for study 1 and study 2 (Table 3). We reiterate that the actual sample size to be used in the trial has been determined primarily by practical considerations, particularly the availability of suitable wards at the participating sites.

Statistical analysis

Study 1 Both non-inferiority and superiority analyses will be conducted. For non-inferiority analyses, if the non-inferiority null hypothesis is to be rejected, the upper limit of the CI around the observed difference should be less than the non-inferiority margin. Multilevel, mixed-effects generalised linear model analyses will be used to construct the 95% CIs that compare effectiveness and safety outcomes between groups. These models will nest patient admissions within wards, treating both as random factors, to account for the clustered nature of these data. Weekend allied health service delivery model provided will be treated as a fixed factor. The distribution of the length of stay outcome will be examined (± transformations as indicated) as a part of model building as these data are commonly skewed. Data from

Table 3 Outcome of power analysis for three outcomes for each study at each site (assuming six wards per site), and for both sites combined

Outcome	Proportion 1 (weekend allied health services)[a]	Proportion 2 (no weekend allied health services)	Single site or both sites combined	Assumed coefficient of variation	Assumed number of patients per study	Power
Proportion of patients who stay longer than their AR-DRG average inlier length of stay	0.40	0.42	Single	0.4	n = 7,000[b]	0.65
Proportion of patients who are readmitted within 28 days	0.10	0.12	Single	0.4	n = 7,000[b]	0.96
Proportion of patients who experience at least one of the adverse events listed	0.10	0.12	Single	0.4	n = 7,000[b]	0.96
Proportion of patients who stay longer than their AR-DRG average inlier length of stay	0.40	0.42	Both	0.4	n = 14,000[b]	0.99
Proportion of patients who are readmitted within 28 days	0.10	0.12	Both	0.4	n = 14,000[b]	>0.99
Proportion of patients who experience at least one of the adverse events listed	0.10	0.12	Both	0.4	n = 14,000[b]	>0.99

AR-DRG, Australian Refined Diagnosis-Related Group.

[a]Baseline proportions based on data drawn from administrative datasets at participating sites covering a 12-month period.

[b]Note that for the single site analyses we assume there will be 7,308 per study, but use only 7,000 in the power analysis to allow for loss of patients during the transition phases between intervention and control conditions and for transfers between study wards. Correspondingly, we use 14,000 when considering the power of both sites combined.

patients who are exposed to both control and intervention conditions within each study will be excluded from the analyses. Analyses will be adjusted for index study ward (first study ward the patient was admitted to during that inpatient episode of care), calendar time using each step in the wedge design as a categorical covariate and for historical data collected for the outcome being examined from each site over the previous 2 years mapped against these steps to account for potential local seasonal variations in the outcomes collected.

Analyses will be conducted at a site-level with preplanned meta-analyses across both sites using patient-level data from phase 1 and separately for phase 2. These meta-analyses will examine the main effect of the usual care weekend allied health service as opposed to the control condition across sites. A site-by-intervention interaction effect will also be examined to determine if there is heterogeneity in results between sites. This is plausible as a dose-response relationship may exist between the amount of resource allocated to the weekend allied health service and the study outcomes. In this study, local data collected prior to the study indicated that Dandenong Hospital allocated approximately four times as much resource to the acute medical and surgical wards participating in this research. Meta-regression will be employed using ward-level data, treating each ward over time as a pre-post intervention study to identify if the labour costs consumed on each ward for provision of the weekend allied health service explains variation in the change in each outcome within ward.

Patients who are exposed to both intervention and control conditions will be excluded from analyses to minimise potential contamination that is made possible by having the transition periods within the stepped wedge design.

Study 2 These analyses will be equivalent to the analyses conducted for study 1; however, non-inferiority analyses will not be conducted for this study.

A grand meta-analysis is planned, pooling results between studies 1 and 2 to examine whether there is a significant study-by-intervention interaction effect. This analysis will examine whether the effect of the stakeholder-driven model of weekend allied health service was significantly different to that of the usual care weekend allied health service delivery model.

Sensitivity analyses will be conducted if there are unit closures (for example, surgical unit closures over the Christmas holiday period) that are likely to affect the caseload on related wards at these times. Data within these cells of the stepped wedge design will be treated as missing in the sensitivity analyses.

Economic analysis The primary economic analysis will be a cost-effectiveness analysis from the hospital perspective that examines the incremental cost per patient admitted. These costs will include the 'total cost' of patients during their admission plus additional costs from an unplanned admission within 28 days. Data from patients who are transferred between wards in different study periods (that is one without access to allied health services, one with access to allied health services) will be excluded from the analyses as will data from patients who remain within the same ward over two or more weekends if they are allowed to access weekend allied health services during one weekend, and not allowed to access allied health services on another. Secondary economic evaluations will examine the incremental cost per difference in clinical outcome for the measures of functional independence (studies 1 and 2) and health-related quality of life (study 2).

Qualitative data analysis Both content and thematic analysis will be used for the analysis of qualitative data collected as a part of this study [38].

Analysis of workforce absenteeism outcome It was initially planned to analyse these data in a similar manner to other outcomes; however, it has become apparent that for some professional groups, staff operate over multiple wards that may be in the intervention or control periods during the study. Hence, we will only compare data collected from the first and last months of each study when all wards within the study are either completely in control or intervention periods.

Deviation from registered trial protocol

There have been two changes to the registered trial protocol. The first change was the withdrawal from both study 1 and study 2 of what was planned to be a third study site. Originally, a third site with five participating wards was planned to be involved and the appropriate ethics approval was gained. The ethics committee at this site retracted this approval due to subsequent opposition from senior medical staff. Project investigators met with these senior medical staff to discuss their concerns; however, agreement could not be reached regarding the issues raised to enable the trial to continue at this site.

The second change was that the broader health network, within which the Western Health (Footscray) site operates, decided to transfer some services from that site to another site within their network. This has resulted in only 5 wards being available to participate in study 2 at this site. This is likely to result in some changes to casemix at the Western Health (Footscray) site during study 2. These changes are planned to take effect in early 2015 when the intervention phase of study 2 is in progress,

thus it was decided to make the changes to the study wards involved from the commencement of phase 2.

Discussion

Disinvestment from health services is becoming a growing priority for health care service providers due to increasing health care expenditure, budget cuts, an increasing burden of chronic disease and ageing in developed nations, and the ongoing introduction of new health technologies that render older technologies obsolete [39-41]. Paradigms for undertaking disinvestment are emerging but current processes rely upon the availability of evidence of effectiveness and cost-effectiveness [39,42,43]. This study uses a novel research design to demonstrate how health services can generate evidence regarding effectiveness, cost-effectiveness and safety during the disinvestment process [21]. The withdrawal of an intended study site due to opposition from senior medical staff despite prior ethics approval has demonstrated a clear barrier to conducting disinvestment research.

Trial status

Recruitment ongoing, anticipated completion June 2015.

Additional file

Additional file 1: SPIRIT 2013 Checklist: recommended items to address in a clinical trial protocol and related documents*.

Abbreviations

ARD-RG: Australian Refined Diagnosis-Related Group; EQ-5D-5 L: European Quality of Life, 5 dimensions; QALY: quality-adjusted life year; TIDieR: Template for Intervention Description and Replication.

Competing interests

The authors declare that they have no competing interests.

Authors' contributions

TPH: led development of research design and analysis plan and overall project management. LOB: assisted development of research design, led development of qualitative data collection methods and collection of qualitative data. DMi: site liaison for Monash Health, assisted with overall project management, led development of qualitative data collection methods and collector of qualitative data across all sites. KAB: led development of quantitative data collection methods, assisted with overall project management and collection of quantitative data across all sites. RH: development of local data collection methods and data collection at Monash Health. DMa: assisted development of overall research design, assisted overall project management and conduct of project at Dandenong Hospital. SP: site liaison for Melbourne Health, assisted with overall project management, assisted development of data collection plan at Melbourne Health. TC: assisted development of overall research design, assisted with overall project management, and conduct of project at Western Health. KM: assisted development of overall research design, assisted with overall project management, and conduct of project at Dandenong Hospital. KP: assisted with overall development of research design and assisted with overall project management. DL: contribution to project management, administration and communications strategy. FM: assisted with development of qualitative data collection approach and assistance with qualitative data collection. MS: assisted with development of quantitative data collection approach at Monash Health and data collection at this site. MG: assisted with development of quantitative data collection approach at Western Health and data collection at this site. LS: site liaison for Melbourne Health, assisted with development of quantitative data approach at Melbourne Health. GJ: assisted with overall project management. ES: assisted with development of overall research design, site liaison for Western Health, assisted with development of quantitative data approach at Melbourne Health, assisted with data collection, assisted with overall project management. All authors have read and approved the trial manuscript and given approval for this version to be published.

Acknowledgements

Funding for this trial has been received from a partnership grant from the National Health and Medical Research Council (NHMRC) Australia (ID 1060696) and from the Department of Health, Victoria. TH is supported by a Career Development Fellowship from the NHMRC (ID 1069758).

Access to trial data

TPH will have access to the final, de-identified trial dataset.

Author details

[1]Allied Health Research Unit, Monash Health and Physiotherapy Department, Monash University, Melbourne, Victoria, Australia. [2]Department of Occupational Therapy, School of Primary Health Care, Faculty of Medicine, Nursing and Health Sciences, Monash University, Frankston, Melbourne, Victoria, Australia. [3]Allied Health, Monash Health, Melbourne, Victoria, Australia. [4]Allied Health, Melbourne Health (Royal Melbourne Hospital), Parkville, Melbourne, Victoria, Australia. [5]Physiotherapy Department, Western Health, Footscray, Melbourne, Victoria, Australia. [6]Health Workforce Branch, Department of Health, Melbourne, Victoria, Australia. [7]Department of Social Work, Monash Health and Monash University, Melbourne, Victoria, Australia.

Received: 10 January 2015 Accepted: 24 February 2015
Published online: 02 April 2015

References

1. Campbell L, Bunston R, Colangelo S, Kim D, Nargi J, Hill K, et al. The provision of weekend physiotherapy services in tertiary-care hospitals in Canada. Physiother Can. 2010;62(4):347–54.
2. Shaw KD, Taylor NF, Brusco NK. Physiotherapy services provided outside of business hours in Australian hospitals: a national survey. Physiother Res Int. 2012;18(2):115–23.
3. Cusick A, Johnson L, Bissett M. Occupational therapy in emergency departments: Australian practice. J Eval Clin Pract. 2009;15(2):257–65.
4. Beder J. Evaluation research on social work interventions: a study on the impact of social worker staffing. Soc Work Health Care. 2008;47(1):1–13.
5. Cifu DX, Kreutzer JS, Kolakowsky-Hayner SA, Marwitz JH, Englander J. The relationship between therapy intensity and rehabilitative outcomes after traumatic brain injury: a multicenter analysis. Arch Phys Med Rehabil. 2003;84(10):1441–8.
6. Oldmeadow LB, Edwards ER, Kimmel LA, Kipen E, Robertson VJ, Bailey MJ. No rest for the wounded: early ambulation after hip surgery accelerates recovery. ANZ J Surg. 2006;76(7):607–11.
7. Peiris CL, Taylor NF, Shields N. Extra physical therapy reduces patient length of stay and improves functional outcomes and quality of life in people with acute or subacute conditions: a systematic review. Arch Phys Med Rehabil. 2011;92(9):1490–500.
8. Schweickert WD, Pohlman MC, Pohlman AS, Nigos C, Pawlik AJ, Esbrook CL, et al. Early physical and occupational therapy in mechanically ventilated, critically ill patients: a randomised controlled trial. Lancet (London, England). 2009;373(9678):1874–82.
9. Brusco NK, Paratz J. The effect of additional physiotherapy to hospital inpatients outside of regular business hours: a systematic review. Physiother Theory Pract. 2006;22(6):291–307.
10. Brusco NK, Shields N, Taylor NF, Paratz J. A Saturday physiotherapy service may decrease length of stay in patients undergoing rehabilitation in hospital: a randomised controlled trial. Aust J Physiother. 2007;53(2):75–81.
11. DiSotto-Monastero M, Chen X, Fisch S, Donaghy S, Gomez M. The efficacy of seven days per week inpatient admissions and rehabilitation therapy. Arch Phys Med Rehabil. 2012;93(12):2165–9.
12. Hakkennes S, Lindner C, Reid J. Implementing an inpatient rehabilitation Saturday service is associated with improved patient outcomes and

facilitates patient flow across the health care continuum. Disabil Rehabil. 2014;(ePub doi:10.3109/09638288.2014.939772)
13. Peiris CL, Shields N, Brusco NK, Watts JJ, Taylor NF. Additional Saturday rehabilitation improves functional independence and quality of life and reduces length of stay: a randomised controlled trial. BMC Med. 2013;11:1–11.
14. Victorian Public Health Sector (Health Professionals, Health and Allied Services, Managers & Administrative Officers) Enterprise Agreement 2011-2015. http://www.fwc.gov.au/documents/agreements/fwa/AE896737.pdf. Accessed 1st November 2015.
15. Brue SL. Retrospectives: the law of diminishing returns. J Econ Perspect. 1993;7(3):185–92.
16. Haines TP, Kuys S, Clarke J, Morrison G, Bew P. Dose-response relationship between physiotherapy resource provision with function and balance improvements in patients following stroke: a multi-centre observational study. J Eval Clin Pract. 2011;17(1):136–42.
17. David C, Price N, Price T, Sheeran T, Mulherin D. Impact of weekend physiotherapy delivery on the throughput of rheumatology inpatients: feasibility study. Physiotherapy. 2003;89(1):25–9.
18. Hughes K, Kuffner L, Dean B. Effect of weekend physical therapy treatment on postoperative length of stay following total hip and total knee arthroplasty. Physiother Can. 1993;45(4):245.
19. Ponto JM, Berg W. Social work services in the emergency department: a cost-benefit analysis of an extended coverage program. Health Soc Work. 1992;17(1):66–73.
20. Brusco NK, Watts JJ, Shields N, Taylor NF. Are weekend inpatient rehabilitation services value for money? An economic evaluation alongside a randomized controlled trial with a 30 day follow up. BMC Med. 2014;12(1):89.
21. Haines T, O'Brien L, McDermott F, Markham D, Mitchell D, Watterson D, et al. A novel research design can aid disinvestment from existing health technologies with uncertain effectiveness, cost-effectiveness, and/or safety. J Clin Epidemiol. 2014;67(2):144–51.
22. Hoffmann TC, Glasziou PP, Boutron I, Milne R, Perera R, Moher D, et al. Better reporting of interventions: template for intervention description and replication (TIDieR) checklist and guide. BMJ. 2014;348:g1687.
23. Roland M, Torgerson DJ. Understanding controlled trials: what are pragmatic trials? BMJ. 1998;316(7127):285.
24. Craig P, Dieppe P, Macintyre S, Michie S, Nazareth I, Petticrew M. Developing and evaluating complex interventions: the new Medical Research Council guidance. BMJ. 2008;337:a1655.
25. Hasson F, Keeney S, McKenna H. Research guidelines for the Delphi survey technique. J Adv Nurs. 2000;32(4):1008–15.
26. Organisation for Economic Co-operation and Development (2013), Health at a Glance 2013: OECD Indicators, OECD Publishing. http://dx.doi.org/10.1787/health_glance-2013-en. Accessed 1st November 2014
27. Victorian health policy and funding guidelines 2012-2013. http://www.health.vic.gov.au/pfg/. Accessed 1st November 2015.
28. Kossovsky MP, Sarasin FP, Perneger TV, Chopard P, Sigaud P, Gaspoz JM. Unplanned readmissions of patients with congestive heart failure: do they reflect in-hospital quality of care or patient characteristics? Am J Med. 2000;109(5):386–90.
29. Stanley K. Design of randomized controlled trials. Circulation. 2007;115(9):1164–9.
30. Draper M, Cohen P, Buchan H. Seeking consumer views: what use are results of hospital patient satisfaction surveys? Int J Qual Health Care. 2001;13(6):463–8.
31. Shah S, Vanclay F, Cooper B. Improving the sensitivity of the Barthel Index for stroke rehabilitation. J Clin Epidemiol. 1989;42(8):703–9.
32. EuroQol Group. EuroQol - a new facility for the measurement of health-related quality of life. Health Policy (Amsterdam, Netherlands). 1990;16(3):199.
33. Ran C, Wen S, Yonghe W, Honglu M. A glimpse of community-based rehabilitation in China. Disabil Rehabil. 1992;14(2):103–7.
34. Ho CH. Stopping rules for clinical trials implicitly incorporating safety information. Biometrical J. 1991;33(7):817–27.
35. Cohen J. Statistical power analysis for the behavioral sciences: Lawrence Erlbaum Associates, New Jersey; 1988
36. Snapinn SM. Noninferiority trials. Curr Control Trials Cardiovasc Med. 2000;1(1):19–21.
37. Hussey M, Hughes J. Design and analysis of stepped wedge cluster randomized trials. Contemp Clin Trials. 2007;28:182–91.
38. Pope C, Ziebland S, Mays N. Analysing qualitative data. Br Med J. 2000;320:114–6.
39. Elshaug AG, Hiller JE, Tunis SR, Moss JR. Challenges in Australian policy processes for disinvestment from existing, ineffective health care practices. Austr New Zeal Health Policy. 2007;4(1):23.
40. Elshaug AG, McWilliams JM, Landon BE. The value of low-value lists. JAMA. 2013;309(8):775–6.
41. Polisena J, Clifford T, Elshaug AG, Mitton C, Russell E, Skidmore B. Case studies that illustrate disinvestment and resource allocation decision-making processes in health care: a systematic review. Int J Technol Assess Health Care. 2013;29(2):174–84.
42. Ibargoyen-Roteta N, Gutiérrez-Ibarluzea I, Asua J. Guiding the process of health technology disinvestment. Health Policy. 2010;98(2):218–26.
43. Joshi NP, Stahnisch FW, Noseworthy TW. Reassessment of health technologies: obsolescence and waste. 2009.
44. Australian Qualifications Framework Council. Australian qualifications framework. 2013. http://www.aqf.edu.au/. Accessed 1st November 2015.

Submit your next manuscript to BioMed Central and take full advantage of:

- **Convenient online submission**
- **Thorough peer review**
- **No space constraints or color figure charges**
- **Immediate publication on acceptance**
- **Inclusion in PubMed, CAS, Scopus and Google Scholar**
- **Research which is freely available for redistribution**

Submit your manuscript at
www.biomedcentral.com/submit

BioMed Central
