# Supplementary material for: Impact of disinvestment from weekend allied health services across acute medical and surgical wards: 2 stepped-wedge cluster randomised controlled trials
Source: PLoS Med. 2017 Oct 31;14(10):e1002412. doi: 10.1371/journal.pmed.1002412 (PMC5663333; doi:10.1371/journal.pmed.1002412)
Supplement: S3 Text — (DOCX) [file pmed.1002412.s006.docx]

**S3 Text – TIDieR criteria**

| **TIDieR criteria** | **Study 1 intervention** | **Study 2intervention** |
| --- | --- | --- |
| Item 1. Brief name: provide the name or a phrase that describes the intervention | Usual care weekend allied health service. | Stakeholder-driven weekend allied health service. |
| Item 2. Why: describe any rationale, theory, or goal of the elements essential to the intervention | Usual care is the prevailing model of care in the research location that in a pragmatic research design serves as an appropriate reference standard. This model of care has developed incrementally over time and has largely been driven by decisions of individual allied health managers in an ‘*ad hoc*’ manner. | A new model of weekend allied health service was developed by managers and staff of participating wards identifying the most important tasks that they felt required completion on weekends that could be undertaken by allied health staff. It was anticipated that by directly engaging with these key stakeholders, a new model of care would be developed that better met the needs of individual wards. |
| Item 3. What (materials)?: describe any physical or informational materials used in the intervention, including those provided to participants or used in intervention delivery or in training of intervention providers | There were no specific materials used beyond those materials ordinarily used by allied health professionals during the week. It was left to the discretion of individual staff what materials they used in their clinical practice. | There were no specific materials used beyond those materials ordinarily used by allied health professionals during the week. It was left to the discretion of individual staff what materials they used in their clinical practice. |
| Item 4. What (procedures)?: describe each of the procedures, activities, and/or processes used in the intervention, including any enabling or support activities | Allied health services included services provided by physiotherapy, occupational therapy, social work, dietetics, speech pathology professionals and allied health assistants. Services delivered were the same as those performed on weekdays, although the intensity of weekend services was lower (fewer hours per ward) than weekday services. Services provided included mobilisation, chest physiotherapy, discharge planning, assessment and prescription of aids and equipment, swallowing assessment, dietary analysis and prescription, and counselling. | Allied health services included services provided by physiotherapy, occupational therapy, social work, dietetics, speech pathology professionals and allied health assistants. Services delivered were the same as those performed on weekdays, although the intensity of weekend services was lower (fewer hours per ward) than weekday services. Priority was given to discharge planning services, though other services could be provided including mobilisation, chest physiotherapy, assessment and prescription of aids and equipment, swallowing assessment, dietary analysis and prescription, and counselling. |
| Item 5. Who provided?: for each category of intervention provider (for example, psychologist, nursing assistant), describe their expertise, background and any specific training given | All allied health professionals had entry-level allied health degrees as a minimum. Orientation of new staff members to the health care organisation and wards that they work on was provided as a part of standard human resources procedures. Allied health assistants do not require formal qualification but all operated under the direction of an allied health professional. | All allied health professionals had entry-level allied health degrees as a minimum. Orientation of new staff members to the health care organisation and wards that they work on was provided as a part of standard human resources procedures. Allied health assistants do not require formal qualification but all operated under the direction of an allied health professional. |
| Item 6. How?: describe the modes of delivery (such as face to face or by some other mechanism, such as Internet or telephone) of the intervention and whether it was provided individually or in a group | Face to face individual patient interaction. | Face to face individual patient interaction. |
| Item 7. Where: describe the type(s) of location(s) where the intervention occurred, including any necessary infrastructure or relevant features | Hospital acute medical/surgical ward environment. | Hospital acute medical/surgical ward environment. |
| Item 8. When and how much?: describe the number of times the intervention was delivered and over what period of time including the number of sessions, their schedule, and their duration, intensity or dose | The frequency of weekend allied health service delivery was presented in Figure 1. The distribution of allied health hours across different professional groups is presented in Supplementary Material 2. | The frequency of weekend allied health service delivery was presented in Figure 1. The distribution of allied health hours across different professional groups is presented in Supplementary Material 2. |
| Item 9. Tailoring: if the intervention was planned to be personalised, titrated or adapted, then describe what, why, when, and how | All weekend allied health services were tailored to the needs of the patients being treated. This was at the discretion of the treating allied health professional based upon their clinical judgement. | All weekend allied health services were tailored to the needs of the patients being treated. This was at the discretion of the treating allied health professional based upon their clinical judgement. |
| Item 10. Modifications: if the intervention was modified during the course of the study, describe the changes (what, why, when, and how) | No modifications made. | No modifications made. |
| Item 11. How well (planned)?: if intervention adherence or fidelity was assessed, describe how and by whom, and if any strategies were used to maintain or improve fidelity, describe them | Patient contact statistics were recorded by allied health professionals through hospital administrative records and were presented in Figure 1. Project research assistants were present each day of the study to clarify to staff which wards were able to have a weekend allied health service and which were not. | Patient contact statistics were recorded by allied health professionals through hospital administrative records and were presented in Figure 1. Project research assistants were present each day of the study to clarify to staff which wards were able to have a weekend allied health service and which were not. |
| Item 12. How well (actual)?: If intervention adherence or fidelity was assessed, describe the extent to which the intervention was delivered as planned | Intervention delivery was presented in Figure 1. A small number of allied health sessions were provided during the control period, though these included clinical exceptions and have been described in the main text of the manuscript. | Intervention delivery was presented in Figure 1. A small number of allied health sessions were provided during the control period, though these included clinical exceptions and have been described in the main text of the manuscript. |
