# Supplementary material for: Impact of disinvestment from weekend allied health services across acute medical and surgical wards: 2 stepped-wedge cluster randomised controlled trials
Source: PLoS Med. 2017 Oct 31;14(10):e1002412. doi: 10.1371/journal.pmed.1002412 (PMC5663333; doi:10.1371/journal.pmed.1002412)
Supplement: S4 Text — Hours of allied health service provision offered under current and newly developed weekend allied health service delivery models at each site. (DOCX) [file pmed.1002412.s007.docx]

**S4 Text – Hours of service by discipline**

Hours of allied health service provision offered under “current” and “newly developed” weekend allied health service delivery models at each site.

|  | Friday* | Saturday | Sunday | Monday* |
| --- | --- | --- | --- | --- |
| “Current” Dandenong Hospital | Nil | 8 hours physical therapy  3 hours occupational therapy  3.5 hours speech pathology  2 hours dietetics  1 hour social work  4 hours allied health assistant | 11 hours physical therapy  3 hours occupational therapy  3 hours speech pathology  2 hours dietetics  1 hour social work  4 hours allied health assistant | Nil |
| “Newly developed” Dandenong Hospital | 8 hours physical therapy  4 hours occupational therapy  4 hours allied health assistant | 3.5 hours physical therapy  2 hours occupational therapy  3.5 hours speech pathology  1 hours dietetics  1 hour social work | 7 hours physical therapy  2 hours occupational therapy  3 hours speech pathology  1 hours dietetics  1 hour social work  4 hours allied health assistant | 4 hours physical therapy  4 hours occupational therapy |
| “Current” Footscray Hospital | Nil | 3.25 hours physical therapy  3.5 hours occupational therapy | 3.25 hours physical therapy | Nil |
| “Newly developed” Footscray Hospital | Nil | 1 hour ICU physical therapist  6 hours transdisciplinary IRS team member  2 hours speech pathology | 1 hour ICU physical therapist | Nil |

* Indicates the Friday and Monday services that were classified as weekend services provided in addition to the normal weekday staffing levels for Friday and Monday services.
