# Supplementary material for: Impact of disinvestment from weekend allied health services across acute medical and surgical wards: 2 stepped-wedge cluster randomised controlled trials
Source: PLoS Med. 2017 Oct 31;14(10):e1002412. doi: 10.1371/journal.pmed.1002412 (PMC5663333; doi:10.1371/journal.pmed.1002412)
Supplement: S5 Text — (DOCX) [file pmed.1002412.s008.docx]

# S5 Text – Clinical exceptions.

## Clinical exceptions at Dandenong Hospital

In this research, there will be periods of time where allied health services are not routinely provided to particular wards. However, allowances have been made for some patients to be seen by allied health staff. These are called **“Clinical Exceptions”.**

A “Clinical Exception” refers to circumstances where the trial treatment protocol is permitted to be violated due to the risk of harm involved.

The criteria that have been established for Dandenong Hospital, the allied health personnel they relate to, and the justification / evidence supporting their inclusion are presented below.

| **Discipline** | **Patient / context description** | **Justification / evidence supporting** |
| --- | --- | --- |
| Social work | When a woman delivers a fully formed and intact fetus over 12 weeks gestation as a still-birth and a social worker is required to organise burial and provide bereavement support. | Local risk assessment |
| Social work | A child protection issue has been identified and a social worker is required to liaise with the Department of Human Services and / or other relevant authorities. | Local risk assessment |
| Speech Pathology | A patient has been fasted pending a swallowing assessment for up to 72 hours and the 72^nd^ hour is scheduled to fall on the weekend (or public holiday). However, use of this clinical exception should not be the standard approach to manage these patients and efforts should be made to ensure these assessments are undertaken on a Thursday or Friday where possible. | NICE guideline [www.nice.org.uk/CG68](http://www.nice.org.uk/CG68) |
| Physiotherapy | Patients who require non-invasive ventilation and have:   - severe exacerbation of chronic obstructive pulmonary disease (defined as pH < 7.35 and relative hypercarbia) - cardiogenic pulmonary oedema and respiratory failure (e.g. PaO2 < 60 mm Hg; SpO2 < 90%) - immunosuppression with acute respiratory failure (e.g. PaO2 < 60 mm Hg; SpO2 < 90%; pH < 7.35 and relative hypercarbia) - respiratory failure following abdominal surgery (can have continuous positive airways pressure or non-invasive ventilation) - lung resection surgery | Keenan S et al. (2011) provides level 1 evidence (meta-analysis of randomised trials) of impact of this intervention in this population on in-hospital mortality and prevention of intubation. |
| Physiotherapy | If a patient has had a fall | It is organisational risk-management policy that physiotherapists review all patients who have had a fall. |
| Dietetics | A patient who is ‘at risk of refeeding syndrome’ AND requires an adjustment to their feeding regimen in light of their blood test results.  To be “at risk” of refeeding syndrome, the patient must either have one or more of the following:   - Body mass index (kg/m^2^) <16 - Unintentional weight loss >15% in the past three to six months - Little or no nutritional intake for >10 days - Low levels of potassium, phosphate, or magnesium before feeding   Or the patient must have two or more of the following:   - Body mass index <18.5 - Unintentional weight loss >10% in the past three to six months - Little or no nutritional intake for >5 days - History of alcohol misuse or drugs, including insulin, chemotherapy, antacids, or diuretics | [www.nice.org.uk/page.aspx?o=cg032](http://www.nice.org.uk/page.aspx?o=cg032).  Mehanna et al 2008. |

## Western Hospital Clinical Exceptions:

**A clinical exception may be authorised for**:

- The provision of non-invasive ventilation by a physiotherapist in:
  - Patients with a severe exacerbation of chronic obstructive pulmonary disease (Type II defined as pH < 7.35 and relative hypercarbia) or cardiogenic pulmonary oedema (Type I respiratory failure defined as PaO_2_ < 60 mm Hg; SpO_2_ < 90%), Level I evidence, Keenan et al., 2011
  - Immunosuppressed patients with Type I or II acute respiratory failure (e.g. PaO_2_ < 60 mm Hg; SpO_2_ < 90%; pH < 7.35 and relative hypercarbia), Level II evidence (Keenan et al., 2011)
  - Treatment of Type I respiratory failure (e.g. PaO_2_ < 60 mm Hg; SpO_2_ < 90%) in abdominal surgery (CPAP/NIV) or lung resection surgery (NIV), Level II evidence (Michelet et al., 2009; Keenan et al., 2011)

**Patients meeting the following criteria may be authorised for an exception and physiotherapy assessment where:**

- - SpO2 < 90% (if not normal for patient) and requiring FiO_2_ ≥ 0.4 with significant deterioration in the past 24 hours (drop in SpO2 > 5% AND/OR doubling of FiO_2_) AND EITHER OR BOTH OF;
    - high clinical suspicion by treating team of sputum plugging (i.e. moist non-productive cough, palpable fremitus; lung white-out with suspected sputum plugging);
    - presence of extensive acute lobar collapse on chest X-ray (where a recent X-ray exists in past 24 hours).

**Patient groups not meeting this criteria**:

- - Inpatients with an exacerbation of COPD requiring Bubble PEP (evidence of lack of efficacy Level II Osadnik et al., 2014)

**Process for escalating a clinical exception in order for a patient on a “no weekend allied health ward” to be seen by allied health.**

NUM or Medical Officer requested allied health on a research ward

**All clinical exceptions must be approved by the AH Director for the patient to be seen by allied health.**

Site After Hours Coordinator decides that case meets clinical exceptions.

AH coordinator rings AH Director

Site After Hours Coordinator decides case doesn’t meet clinical exceptions.

Patient is not seen by allied health

Pati

**References**

1. Keenan SP, Sinuff T, Burns KEA, et al., 2011. Clinical practice guidelines for the use of noninvasive positive-pressure ventilation and noninvasive continuous positive airway pressure in the acute care setting. *Canadian Medical Association Journal* 183(3): E195-E214.
2. Mehanna H, Moledina J, Travis J. 2008. Refeeding syndrome: what it is, and how to prevent and treat it. BMJ. 336(7659): 1495–1498
3. National Institute for Health and Clinical Excellence. Nutrition support in adults Clinical guideline CG32. 2006. [www.nice.org.uk/page.aspx?o=cg032](http://www.nice.org.uk/page.aspx?o=cg032).
4. National Institute for Health and Clinical Excellence. Stroke: the diagnosis and initial management of acute stroke and transient ischaemic attack. 2008. (Clinical guideline 68.) [www.nice.org.uk/CG68](http://www.nice.org.uk/CG68)
