# Supplementary material for: Impact of disinvestment from weekend allied health services across acute medical and surgical wards: 2 stepped-wedge cluster randomised controlled trials
Source: PLoS Med. 2017 Oct 31;14(10):e1002412. doi: 10.1371/journal.pmed.1002412 (PMC5663333; doi:10.1371/journal.pmed.1002412)
Supplement: S6 Text — (DOCX) [file pmed.1002412.s009.docx]

**S6 Text – Power analyses**

Power analysis from Haines TP, O’Brien L, Mitchell D, Bowles KA, Haas R, Markham D, Plumb S, Chiu T, May K, Philip K, Lescai D. Study protocol for two randomized controlled trials examining the effectiveness and safety of current weekend allied health services and a new stakeholder-driven model for acute medical/surgical patients versus no weekend allied health services. Trials. 2015 Apr 2;16(1):133.

**Sample size estimation**

The sample size in this study is governed by the patient throughput on the participating wards over the trial period. Non-inferiority trials do not preclude testing of superiority and can be done so without statistical penalty; hence, we undertook power calculations for studies 1 and 2 from a superiority analysis perspective. Current data from study wards indicates there will be 7,308 patient admissions in total per study per site. We used the approach for conducting power analyses for stepped wedge trials advocated by Hussey and Hughes based upon the Wald statistic. We applied this approach to 3 of our primary outcomes and demonstrated > 90% power in each case for study 1 and study 2 (Table). We reiterate that the actual sample size to be used in the trial has been determined primarily by practical considerations, particularly the availability of suitable wards at the participating sites.

**Outcome of power analysis for three outcomes for each study at each site (assuming six wards per site), and for both sites combined**

| **Outcome** | **Proportion 1 (weekend allied health services)^a^** | **Proportion 2 (no weekend allied health services)** | **Single site or both sites combined** | **Assumed coefficient of variation** | **Assumed number of patients per study** | **Power** |
| --- | --- | --- | --- | --- | --- | --- |
| Proportion of patients who stay longer than their AR-DRG average inlier length of stay | 0.40 | 0.42 | Single | 0.4 | n = 7,000^b^ | 0.65 |
| Proportion of patients who are readmitted within 28 days | 0.10 | 0.12 | Single | 0.4 | n = 7,000^b^ | 0.96 |
| Proportion of patients who experience at least one of the adverse events listed | 0.10 | 0.12 | Single | 0.4 | n = 7,000^b^ | 0.96 |
| Proportion of patients who stay longer than their AR-DRG average inlier length of stay | 0.40 | 0.42 | Both | 0.4 | n = 14,000^b^ | 0.99 |
| Proportion of patients who are readmitted within 28 days | 0.10 | 0.12 | Both | 0.4 | n = 14,000^b^ | >0.99 |
| Proportion of patients who experience at least one of the adverse events listed | 0.10 | 0.12 | Both | 0.4 | n = 14,000^b^ | >0.99 |

AR-DRG, Australian Refined Diagnosis-Related Group.

^a^Baseline proportions based on data drawn from administrative datasets at participating sites covering a 12-month period.

^b^Note that for the single site analyses we assume there will be 7,308 per study, but use only 7,000 in the power analysis to allow for loss of patients during the transition phases between intervention and control conditions and for transfers between study wards. Correspondingly, we use 14,000 when considering the power of both sites combined.
