# Supplementary material for: Impact of disinvestment from weekend allied health services across acute medical and surgical wards: 2 stepped-wedge cluster randomised controlled trials
Source: PLoS Med. 2017 Oct 31;14(10):e1002412. doi: 10.1371/journal.pmed.1002412 (PMC5663333; doi:10.1371/journal.pmed.1002412)
Supplement: S7 Text — Effect size estimates of main and trial-by-site interaction effects from each trial for primary and secondary outcomes. Main effects are interpreted as the impact of being exposed to the no weekend allied health condition compared to the current or newly developed weekend allied health conditions. (DOCX) [file pmed.1002412.s010.docx]

**S7 Text – Analyses unadjusted for monthly ward data from previous 2 years.**

Effect size estimates of main and trial-by-site interaction effects from each trial for primary and secondary outcomes. Main effects are interpreted as the impact of being exposed to the “no weekend” allied health condition compared to the “current” and/or “newly developed” weekend allied health conditions.

|  | Trial 1 | |  | Trial 2 | |  |
| --- | --- | --- | --- | --- | --- | --- |
|  | Main effect | Intervention-by-site interaction | ICC* | Main effect | Intervention-by-site interaction | ICC* |
| **Primary** |  |  |  |  |  |  |
| Length of stay (days) | 1·34 (0·89 to 1·80), p<0·001^€^ | -0·14 (-0·73 to 0·46), p=0·66 | S:0·0003  W:0·02  E:0·91 | -1·55 (-1·99 to -1·11), p<0·001^€^ | 0·25 (-0·39 to 0·90), p=0·45 | S:0·0003  W:0·03  E:0·88 |
| Length of stay (log transformed) | 0·10 (0·04 to 0·16), p=0.001 | -0·09 (-0·17 to -0.014), p=0·02 | S: 0·0002  W:0·06  E:0·90 | -0·14 (-0·20 to -0·07), p<0·001 | -0·03 (-0·13 to 0·06), p=0·47 | S:0·0003  W:0·07  E:0·88 |
| Proportion of patients staying longer than expected | 0·01 (-0·01 to 0·04), p=0·30 | -0·02 (-0·06 to 0·02), p=0·30 | S:0·82  W:0·85 | -0·02 (-0·05 to 0·00), p=0·08 | -0·00 (-0·04 to 0·04), p=0·86 | S:0·84  W:0·88 |
| Proportion with an unplanned readmission within 28 days | 0·01 (-0·01 to 0·03), p=0·19 | -0·04 (-0·07 to -0·02), p<0·001^€^ | S:^£^  W:0·38 | -0·01 (-0·02 to 0·01), p=0·62 | -0·02 (-0·05 to 0·01), p=0·18 | S: ^£^  W:0·53 |
| Proportion of patients with any adverse event | 0·01 (-0·01 to 0·03), p=0·33 | -0·02 (-0·04 to 0·01), p=0·15 | S: ^£^  W:0·71 | -0·02 (-0·04 to -0·00), p=0·11 | 0·04 (0·01 to 0·07), p=0·01 | S: ^£^  W:0·66 |
| **Secondary** |  |  |  |  |  |  |
| Proportion of patients discharged to aged care: | 0·00 (-0·00 to 0·01), p=0·28 | -0·00 (-0·01 to 0·01), p=0·54 | S:0·30  W:0·65 | -0·00 (-0·01 to 0·01), p=0·81 | 0·00 (-0·01 to 0·01), p=0·99 | S: 0·12  W:0·62 |
| Cost to the health care system per admission | 1810 (1094 to 2525), p<0·001 | 769 (-168 to 1706), p=0·11 | S: ^£^  W:0·013  E:0·76 | -2431 (-3166 to -1696), p<0·001^€^ | -163 (-1273 to 947), p=0·77 | S: ^£^  W:0·005  E:0·88 |
| Proportion of patients discharged on a Saturday or Sunday | 0.01 (-0.01 to 0.03), p=0.33 | 0.02 (-0.01 to 0.05), p=0.24 | S: ^£^  W:0·43 | -0.02 (-0.05 to -0.00), p=0.03^€^ | -0.01 (-0.04 to 0.02), p=0.69 | S: ^£^  W:0·47 |

*ICCs derived from mixed effects generalized linear models partitioned at the site level (S), ward (W), +/- patient episode (E) levels.

^£^ Denotes ICC values less than 0·0001.

^€^  Denotes statistical significance (two tailed p<0·05)
